# Water Use, Leaf Cooling and Carbon Assimilation Efficiency of Heat Resistant Common Beans Evaluated in Western Amazonia

**DOI:** 10.3389/fpls.2021.644010

**Published:** 2021-11-29

**Authors:** Juan Carlos Suárez, Milan O. Urban, Amara Tatiana Contreras, Jhon Eduar Noriega, Chetan Deva, Stephen E. Beebe, José A. Polanía, Fernando Casanoves, Idupulapati M. Rao

**Affiliations:** ^1^Facultad de Ingeniería, Programa de Ingeniería Agroecológica, Universidad de la Amazonia, Florencia, Colombia; ^2^Facultad de Ingeniería, Programa de Maestría en Sistemas Sostenibles de Producción, Universidad de la Amazonia, Florencia, Colombia; ^3^Centro de Investigaciones Amazónicas CIMAZ Macagual César Augusto Estrada González, Grupo de Investigaciones Agroecosistemas y Conservación en Bosques Amazónicos-GAIA, Florencia, Colombia; ^4^International Center for Tropical Agriculture (CIAT), Cali, Colombia; ^5^Climate Impacts Group, School of Earth and Environment, Institute for Climate and Atmospheric Science, University of Leeds, Leeds, United Kingdom; ^6^Departamento de Biología Molecular de Plantas, Instituto de Biotecnología, Universidad Nacional Autónoma de México, Cuernavaca, Mexico; ^7^CATIE - Centro Agronómico de Investigación y Enseñanza, Turrialba, Costa Rica

**Keywords:** acid soil, climatology, common bean, heat resistance, leaf cooling, partitioning, water spender, water saver

## Abstract

In our study, we analyzed 30years of climatological data revealing the bean production risks for Western Amazonia. Climatological profiling showed high daytime and nighttime temperatures combined with high relative humidity and low vapor pressure deficit. Our understanding of the target environment allows us to select trait combinations for reaching higher yields in Amazonian acid soils. Our research was conducted using 64 bean lines with different genetic backgrounds. In high temperatures, we identified three water use efficiency typologies in beans based on detailed data analysis on gasometric exchange. Profligate water spenders and not water conservative accessions showed leaf cooling, and effective photosynthate partitioning to seeds, and these attributes were found to be related to higher photosynthetic efficiency. Thus, water spenders and not savers were recognized as heat resistant in acid soil conditions in Western Amazonia. Genotypes such as BFS 10, SEN 52, SER 323, different SEFs (SEF 73, SEF 10, SEF 40, SEF 70), SCR 56, SMR 173, and SMN 99 presented less negative effects of heat stress on yield. These genotypes could be suitable as parental lines for improving dry seed production. The improved knowledge on water-use efficiency typologies can be used for bean crop improvement efforts as well as further studies aimed at a better understanding of the intrinsic mechanisms of heat resistance in legumes.

## Introduction

The common bean (*Phaseolus vulgaris* L.) is the most important legume in people’s diet in the tropics of Latin America and East Africa (Beebe et al., 2008; Andrade et al., 2016; Polanía et al., 2016c; Asfaw et al., 2017). The majority of common bean production is from developing countries by small-scale producers (Polania et al., 2016a), where it is affected by climatic variations that limit its production (Beebe et al., 2013; Polania et al., 2016b). In an intermediate greenhouse gas emissions scenario, global average temperatures are predicted to increase by 2°C between 2041 and 2060 (IPCC, 2021), which may result in a loss of 50% of the area currently planted by 2050 (Beebe et al., 2011; Rippke et al., 2016; Rao et al., 2017).

Heat stress in bean cultivation generates a series of irreversible damages in the metabolism and development of plants (Omae et al., 2007, 2012; Porch and Hall, 2013; Sofi and Maduraimuthu, 2017; Suárez et al., 2020), according to observations made across different environments (Costa et al., 2000; Omae et al., 2012; Suárez et al., 2018a, b, 2020). At a physiological level, several processes are affected (Wentworth et al., 2006), photosynthesis is highly sensitive to high temperatures (Mathur et al., 2014) generating a decrease in the photosynthetic absorption of CO_2_ (Perdomo et al., 2016); the leaf senescence and expansion is reduced (Wahid et al., 2007) as well as the grain yield (Suárez et al., 2020).

Mainly, the rate of photosynthesis is affected by stomatal closure that limits the diffusion of CO_2_ from the stomata, through intercellular spaces and carboxylation sites through the resistance in mesophyll (Bigras, 2005; Cardona-Ayala et al., 2014), restricting growth and dry matter accumulation (Chaves and Oliveira, 2004; Amsalu et al., 2014). At the same time, the way they are affected is related to physiological responses such as transpiration, respiration, antioxidant activity, light absorption and capture (Lawlor and Cornic, 2002; Silva et al., 2010; Falqueto et al., 2017), electron transport capacity (*J*_max_), maximum carboxylation rate (*V*_cmax_), (Dreyer et al., 2001; Yamori et al., 2006, 2008), ATP synthesis and the RuBP regeneration capacity (Wise et al., 2004).

Heat is one of the main constraints on plant adaptability and productivity (Omae et al., 2012), so plant water status is paramount under high temperature conditions as plants attempt to stabilize water content in their tissues (Fahad et al., 2017). Mechanisms of heat tolerance identified in plants include canopy acclimatization to a gradual increase in temperature (Hong et al., 2003), decreasing the thickness of the leaves (Crawford et al., 2012), and/or by increasing the stomatal conductance (*g_s_*), they sustain the diffusion of CO_2_ in the leaves, improving cooling from transpiration (Porch and Hall, 2013). In addition, some plants have the ability to maintain or increase instantaneous water use efficiency (*WUE*) by maintaining or increasing photosynthesis (Sofi and Maduraimuthu, 2017), mainly due to an overall improvement in the rate of carboxylation and its relative rate to oxygenation, by increasing the amounts of Rubisco and/or its specificity for CO_2_ (Parry et al., 2005; Galmes et al., 2014).

Water use efficiency (*WUE*) has been established as an important attribute that describes the efficiency of plants to use available water for carbon sequestration (Bramley et al., 2013). With rising temperatures and heat stress in the future, increasing *WUE* is vital for sustainable production (Farooq et al., 2019). Although *WUE* is a complex phenotypic trait (Araus et al., 2002; Easlon et al., 2014), it has been proposed that some plant traits can be based on the “water-saving” isohydric plant model or the “water-spending” anisohydric plant model (Blum, 2015). Polania et al. (2016a) identified water-saving bean genotypes that have the characteristic of better instantaneous *WUE* – producing more with less water and less gas exchange, unlike water-spending genotypes that exhibit better effective use of water (*EUW*) by maximizing water capture for more production with better gas exchange. However, these results were generated in lysimeter-like tubes, not in the field conditions.

Throughout its evolution and domestication, the common bean eventually formed two cultivated gene pools, the Mesoamerican gene pool and the Andean gene pool (Debouck and Smartt, 1995), originating in Central and South America (Gepts and Debouck, 1991). The genetic structure of the common bean has been frequently reviewed, because during its global domestication there have been changes in its morphological characteristics, including enlargement of seeds and leaves, alterations in growth habits and photoperiodic response, as well as changes in color and seed coat, leading to an increase in its genetic diversity that has allowed it to develop optimal responses under stress conditions (Singh et al., 1991; Long and Bernacchi, 2003; Mcclean et al., 2004). In this regard, the adverse effects of heat stress on common beans can be likely mitigated through the development of heat-resistant genotypes (Wahid et al., 2007). Understanding the mechanisms and/or physiological strategies of plants at high temperatures is necessary to develop breeding strategies to improve their adaptability (Beebe, 2012; Omae et al., 2012). The identification of heat-resistant bean lines can help to maintain production under conditions of increased temperature as a result of a climate change (Beebe et al., 2011; Beebe, 2012). In this regard, the objective for the present study was to identify the relationship between *WUE* as a high temperature resistance mechanism as well as identifying superior advanced lines (genotypes) of common beans with better heat resistance that contributes to better physiological performance and grain yield under heat stress. The study provides novel information on the physiological behavior and response mechanisms to high temperatures in different common bean genotypes in Western Amazonia. Results and suggestions can be used for crop improvement efforts and for studies aimed at a better understanding of the mechanisms of heat stress resistance in beans.

## Materials and Methods

### Experimental Site and Meteorological Conditions

**Figure 1:**
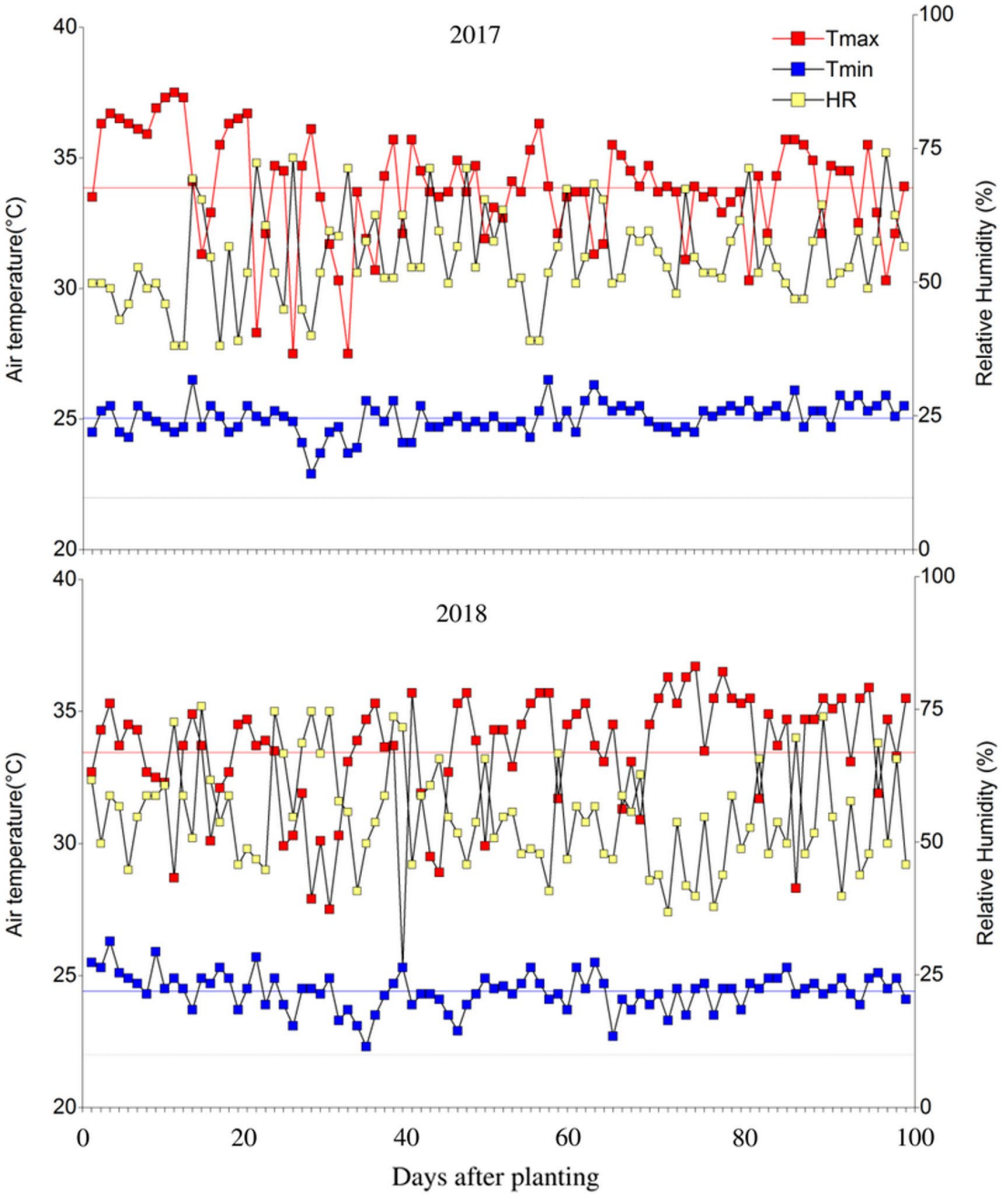
Distribution of maximum/minimum temperatures and relative humidity during the crop growing period under greenhouse conditions at Centro de Investigaciones Amazónicas CIMAZ Macagual, Universidad de la Amazonia, Colombia in two seasons (2017) and (2018). The black line is a mean temperature of 22°C. Red and blue line means the average of the maximum and minimum air temperature, respectively. 2017: *Tair*_max_: 33.86±0.22°C, *Tair*_min_: 25.03±0.06°C, RH: 56.19±0.94%. 2018: *Tair*_max_: 33.45±0.24°C, *Tair*_min_: 24.41±0.07°C, RH: 55.32±1.02%.

The evaluation of the adaptation of bean materials was carried out at the Centro de Investigaciones Amazónicas CIMAZ Macagual, Universidad de la Amazonia (1°37’N and 75°36’W), located in Florencia, Caquetá (Colombia). Located in the moist climate of the tropical rainforest ecosystem, it exhibits an average annual precipitation of 3,800mm, 1700h of sunshine year^−1^, an average temperature of 25.5°C, and a relative humidity of 84%. Under greenhouse conditions the mean maximum and minimum temperatures were 33.8°C and 24.6°C, respectively, the mean daily temperature was 28.5°C and the mean relative air humidity was 56% (Figure 1). These evaluations were conducted in two periods [(1) October 2017 to January 2018; (2) August to November 2018] which corresponded to the driest part of the year and the highest air temperature compared to the other months of the year.

### Plant Material and Experimental Design

A total of 64 bean genotypes were used in this study (Supplementary Table 1). The list include 32 advanced lines of *Phaseolus vulgaris* L. (ALB 348, BFS 10, BFS 35, DAB 295, DAB 525, ICA QUIMBAYA, SCR 40, SCR 45, SCR 56, SEN 136, SEN 52, SEN 70, SER 125,SER 271, SER 316, SER 323, SER 324, SER 48, SER 16, SMC 232, SMN 65, SMN 68, SMN 99, SMR 101, SMR 139, SMR 173, SMR 174, SMR 175, SMR 39, SXB 412, TIO CANELA 75, VAX 1), four Mesoamerican interspecific lines of *P. vulgaris × P. coccineus* (ALB 351, ALB 352, BFS 123, BFS 142), eleven Andean interspecific lines of *P. vulgaris ×* (*P. vulgaris × P. coccineus*; RRA 3, RRA 31, RRA 57, RRA 60, RRA 69, RRA 78, RRA 80, RRA 81, RRA 93, RRA 103, RRA 124), one interspecific line from *P. vulgaris × P. acutifolius* (SER 212), 11 interspecific lines from *P. vulgaris × P. acutifolius × P. coccineus* (SEF 1, SEF 10, SEF 12, SEF 16, SEF 40, SEF 42, SEF 46, SEF 49, SEF 70, SEF 71, SEF 73), one Mesoamerican type with interspecific genes, generated through crossing [(ALB 74 *×* INB 841)F_1_ *×* RCB 593)], and four interspecific lines from *P. vulgaris × P. coccineus* which resulted in a group of Mesoamerican gene pool lines [ALB 121, ALB 191, ALB 210, ALB 60; F_5:6_ generation of SER16 *×* (SER16 *×* G35346-3Q); Butare et al., 2011]. The genotypes tested belong to the Mesoamerican or Andean gene pool and include elite and advanced lines for drought, aluminum resistance, heat, high micronutrient content and other desirable attributes that can improve grain production or market price. The ALB lines (small red kidney, black kidney) were developed for improved adaptation to drought and aluminum toxicity in acidic soil. The BFS (small red) lines have been developed to improve adaptation to low soil fertility and drought. The DAB (red mottled, red-pink) lines have been developed to improve adaptation to drought. The RRA (various colored) lines have been developed to improve resistance to root rots caused by *Pythium* and *Sclerotium*. The SEF, SER and SCR (small red), SEN (small black), SXB (cream) lines have been developed for improved adaptation to drought and heat. The SMC (various colored), SMN (black), SMR (red) lines have been developed to improve tolerance to drought with a high mineral (Fe) content in seed. The VAX (cream striped) line is sensitive to aluminum toxicity. The TIO CANELA 75 (small red kidney) commercial cultivar is recognized as drought sensitive. The ICA QUIMBAYA commercial cultivar is resistant to aluminum. Details on each line in terms of commercial class, gene pool, growth habit, classification and genetic background are provided in Supplementary Table 1. The morphophysiological data are provided in Supplementary Table 2.

A completely randomized plot design with three replications was used. Each experimental unit (advanced bean lines) consisted of a plot sown with three rows of two meters long, with a distance between rows of 0.6m and a spacing between plants of 15cm (equivalent to 11 plants m^−2^). The seeds were planted in a soil with an effective depth of 80cm of soil profile. The water availability was maintained at a field capacity during the whole experiment. It is a clay loam soil (Oxisol) with bulk density values that ranged between 1 and 1.3gcm^−3^, pH values that ranged from pH 4.1 to 5.2, with a mean organic carbon content of 1.35%, available P content (Bray-II) of 2.58mgkg^−1^, saturation of total bases of 7.1% (Ca: 0.38 cmol kg^−1^, Mg: 0.1 cmol kg^−1^, K: 0.14 cmol kg^−1^, Na: 0.1 cmol kg^−1^, total bases: 0.8 cmol kg^−1^), a cation exchange capacity of 11.3 cmol kg^−1^, and an exchangeable aluminum content of 6.3 cmol kg^−1^ with 73.4% of Al saturation.

### Gas Exchange Parameters and Photosynthetic Light- and CO_2_-Response Curves of Common Bean Lines

The gas exchange parameters at leaf level, such as stomatal conductance (*g_s_*, mmol H_2_O m^−2^ s^−1^), transpiration rate (*E*, mmol H_2_O m^−2^ s^−1^), instantaneous water use efficiency (mmol CO_2_ mol^−1^ H_2_O), sub-stomatal CO_2_ concentration (*C*_i_, μmol mol^−1^) were measured using an infrared gas analyzer CIRAS-3 Portable Photosynthesis System (PP Systems Inc. Amesbury, MA, United States) at a partial CO_2_ concentration of 400ppm under artificial photosynthetic active radiation (*PAR*) following the steps outlined by Suárez et al. (2018a,b), where the corresponding air temperature range at the time of the gas exchange measurements was 27.2 to 34.4°C. The stomatal limitation value (*g_lim_*) was calculated using the following formula: g_lim_=1− (C_i_/C_a_), according to Yin et al. (2006). With the difference between the air temperature and the leaf temperature (measured by the CIRAS-3 Portable Photosynthesis System) we calculated the leaf temperature differential (*LTD)*. Gas exchange measurements were taken between 08:00 and 11:00h (solar time) on three fully developed leaves (located between the seventh and ninth leaf developed from the base of the plant) of each plant with three independent replications per genotype, at each monitoring period. The measurements of leaf gas exchange were made at the stage (later R7) when 50% of the pods have reached final length, which corresponds to a period of 50 to 70days after sowing, according to the BBCH scale for bean growth (BBCH 75; Meier, 2001).

The photosynthetic response curves to *PAR* intensity (henceforth, *A/PAR*) were generated by increasing *PAR* in ten steps from 0 to 2,000μmolm^−2^ s^−1^. Initially, the environmental conditions to which the leaves were exposed to in the CIRAS chamber were as follows: vapor pressure deficit (*VPD)* between 1.0 and 1.5kPa, leaf temperature of 25°C, and a partial concentration of CO_2_ of 50ppm for 5min to allow the stomata to open; subsequently *A/PAR* curves were obtained at a partial concentration of CO_2_ of 400ppm. In order to determine the photosynthetic limitations of common bean that result from the microclimatic conditions, the above data were used to calculate different parameters derived from the slope of the initial linear portion of the *A/PAR* curve (Bauerle et al., 2006): light-saturated maximum net carbon assimilation rate (*A*_max_, μmol CO_2_ m^−2^ s^−1^), light compensation point (*LCP*, μmol m^−2^ s^−1^), dark respiration rates (*R*_d_, μmol CO_2_ m^−2^ s^−1^), light saturation point (*LSP*, μmol m^−2^ s^−1^), and apparent quantum efficiency (*Φ*_PAR_, μmol CO_2_ μmol protons^−1^).

Photosynthetic assimilation response curves to internal CO_2_ concentration (henceforth, *A/C*_i_) were made at a saturating light level of 1,300μmolm^−2^ s^−1^ (based on the *A/PAR* curves), at 25°C and ambient O_2_ concentration following the recommendations of Long and Bernacchi (2003). Measurements were initiated at a partial concentration of CO_2_ of 400ppm, which was gradually decreased to 50ppm and subsequently increased in 15 steps up to 1,600ppm of partial concentration of CO_2_ (Martins et al., 2013). Leakage errors were corrected by measuring the CO_2_ response curves in dead leaves following the recommendations of Flexas et al. (2007). Different parameters derived from each *A/C*_i_ curve were determined including maximum rate of ribulose-1,5-bisphosphate carboxylase/oxygenase (RuBisCO), carboxylation (*V*_cmax_), maximum rate of electron transport driving regeneration of ribulose-1, 5-bisphosphate (RuBP; *J*_max_), and leaf respiration under light conditions (*R*_D_).

### Calculation of Stress Index in a High Temperature Environment

To evaluate the agronomic yield upon high temperature environment, destructive sampling (85–90 DAS) was carried out in the central row of each plot, the pods of the harvested plants were threshed, and the grains were cleaned and oven-dried to determine their yield (kgha^−1^). Because in this study, control yields (yield not influenced by heat) were not available, the response of individual genotypes to heat stress in Western Amazonia was determined using three steps calculating genotype stress index (*GSI*). *GSI* in this sense serves the same as other stress indices, to eliminate the effect of intra- and inter-genotypic variability by showing the individual genotype yield in the context of the population median. The first step was to calculate yield reduction (YR) of each individual genotype per experiment [YR=Y_j_/Ÿ (%), where Y_j_=mean yield of individual genotype *_j_*, Ÿ=geometric mean of the whole experiment]. The YR serves as a first standardization of obtained genotype yield toward the geometric yield from the whole individual experiment (year repetition). The second step was to calculate general stress intensity index (SII) as a second data standardization using ratio of geometric means from all (in our study two) years of experiments [SII=1− (Ÿ_j_/Ÿ_i_), where Ÿ_j_ and Ÿ_i_ are geometric yields of all genotypes used in all experiments]. The third step was to calculate *GSI* for each particular genotype used in the study. The *GSI* was calculated using the following equation: *GSI*={1−[Ÿ (YR_*x*1_, YR_*x*2_)]}/SII, where Ÿ is the geometric mean, YR_*x*1_ or _*x*2_ is the yield change per genotype X in first (1) or second (2) year, and SII is general stress intensity index.

### Statistical Analysis

The Michaelis–Menten hyperbolic constant was used to adjust the *A/PAR* curves; the parameters *A*_max_, *LSP*, *LCP*, *R*_d_, and *Φ*_PAR_ were calculated following the equations described in Lobo et al. (2013). The model created by Farquhar et al. (1980) (the ‘FvCB model’) was used to evaluate the *A/C*_i_ curve and to estimate *V*_cmax_, *J*_max_, and *R*_D_ using the “*plantecophys”* package in R (Duursma, 2015). Pearson’s and Spearman’s correlation coefficients were calculated in order to determine significant relationships between variables, which were visualized in a string diagram. To determine the genotypes that best responded to high temperature conditions a scatterplot was used. Variables that affect grain yield (Assimilation *A*, stomatal conductance *gs* and leaf temperature depression *LTD*), or Transpiration (*E*) were plotted on the X-axis. As the dependent variable, grain yield was plotted on the Y-axis. Four quadrants were visualized by plotting the corresponding averages on each axis.

Due to the non-linear behavior of *A* the model adjustment was carried out using linear segment models with two segments given by the following function $V_{s}=\beta_{0}+\left(\beta_{1}x\right)I\left(x<\gamma \right)+\left(\beta_{2}x\right)I\left(x>\gamma \right)$ where $\beta_{0}$ is the intercept, $\beta_{1}$ is the slope of the first segment, γ is the point at which the segments join, and $\beta_{2}$ is the slope of the second segment. The value obtained from the point at which the segments joined, a linear mixed model (LMM) to perform a covariance analysis using genotype as a class variable and ambient temperature as a covariate for *g_s_* and *A* as response variables was prepared. This point corresponds to the maximum ambient temperature value at which the *A* took a linear segment direction. For each genotype we calculated the mean expected value for responses (*g_s_* and *A*) when temperature was 27.74°C. With these estimated values we performed a scatter plot of *A* against *gs* and included a label for each genotype.

Bean genotypes were grouped using all physiological and bean yield variables using cluster analysis for those genotypes that have higher photosynthetic efficiency in relation to instantaneous *WUE* values as well as those that had higher heat tolerance allowing to maintain bean yield in advanced common bean lines. With the conformed bean genotypes typologies, a principal component analysis (PCA) was also performed and the effect of the leaf temperature depression on the different physiological mechanisms to maintain a higher photosynthetic efficiency was tested by means of a Monte Carlo permutation test. The ANOVA using LMM was adjusted to analyze the effect of the fixed factor (Genotype). Plots associated with genotypes within the monitoring period (repeated measurements) were included as random effects. The assumptions of normality and homogeneity of variance were evaluated using an exploratory residual analysis. Differences between genotypes were analyzed with Fisher’s post hoc LSD test with a significance of *α*=0.05. The LMM were made using the *lme* function in the *nlme* package, the cluster analysis, PCA and the graphic outputs were made in the packages “*ade4*,” “*ggplot2*,” “*factoextra*” and “*corrplot*” in the R language software, version 3.4.4 (R Development Core Team, 2019), by the interface in InfoStat (Di Rienzo et al., 2019).

## Results

### Relationships Between Physiological Variables and Grain Yield With High Temperature

The high air temperature had a negative effect on common bean genotypes by decreasing carbon assimilation (*A, r*=−0.42, *p*<0.001 *r*=coefficient of correlation) due to the reduction in stomatal conductance (*g_s_, r*=−0.75, p<0.001) and substomatal CO_2_ concentration (*C_i_*, *r*=−0.57, *p*<0.001) which resulted in lower grain yield (*GY*, Table 1). Likewise, when the air temperature increases, some genotypes increase *WUE* (r=0.63 between *LTD* and *WUE p*<0.001), generating processes of stomatal limitation to photosynthesis (*r*=0.60 between *LTD* and *g_lim_ p*<0.001). For an adequate grain yield, carbon fixation must be high (*r*=0.51, *p*<0.001 between *A* and *GY*), increasing *gs*, resulting in a transpiration cooling mechanism (*r*=0.36, *p*<0.001 between *A* and *g_s_*; *r*=0.51, *p*<0.001 between *A* and *E*, respectively). Furthermore, this characteristic negatively affects the efficient use of water (*r*=−0.88 between *E* and *WUE p*<0.001). There were other characteristics such as *LSP* which was positively related to both *GY* and *A*, as well as to *E*.

**Table 1:** Correlation coefficients (r) between grain yield (*GY*), assimilation (*A*), leaf temperature depression (*LTD*), transpiration (*E*) and stomatal limitation (g_lim_) values and physiological traits at the leaf level of 64 genotypes of bean grown under conditions of high temperature.

Variables	Variables				
	*LTD*	*A*	*GY*	*E*	*g_lim_*
Grain Yield (*GY*, kg ha^−1^)	−0.21	0.51^***^		0.35^***^	−0.15
Assimilation (*A*, μmol CO_2_ m^−2^ s^−1^)	−0.42^***^		0.51^***^	0.51^***^	−0.09
Light saturation point (*LSP*, μmol m^−2^ s^−1^),	−0.2	0.83^***^	0.48^***^	0.28^*^	0.06
Dark respiration rates (*R*_d,_ μmol CO_2_ m^−2^ s^−1^)	−0.32^**^	0.18	0.06	0.23	−0.17
Light compensation point (*LCP*, μmol m^−2^ s^−1^)	−0.26^*^	0.19	0.12	0.2	−0.11
Apparent quantum efficiency (*Φ*_PAR,_ μmol CO_2_ μmol protons^−1^)	0.18	−0.83^***^	−0.42^***^	−0.28^*^	−0.07
Maximum rate of ribulose-1, 5-bisphosphate carboxylase/oxygenase (RuBisCO) carboxylation (*V*_cmax,_ μmol CO_2_ m^−2^ s^−1^)	−0.1	0.2	0.07	0.15	0.03
Maximum rate of electron transport driving regeneration of ribulose-1, 5-bisphosphate (RuBP) (*J*_max,_ μmol CO_2_ m^−2^ s^−1^)	0.08	0.24	−0.03	−0.07	0.2
Leaf respiration under light conditions (*R*_D,_ μmol CO_2_ m^−2^ s^−1^)	−0.09	0.04	−0.11	0.07	−0.1
Stomatal conductance (gs, mmol H_2_O m^−2^ s^−1^),	−0.75^***^	0.36^***^	0.31^**^	0.94^***^	−0.91^***^
Sub-stomatal CO_2_ concentration (*C*i, μmol mol^−1^),	−0.57^***^	−0.04	0.11	0.75^***^	−0.95^***^
Photosynthetic water use efficiency (*WUE*, mmol CO_2_ mol^−1^ H_2_O)	0.63^***^	−0.11	−0.18	−0.88^***^	0.92^***^
Transpiration rate (*E*, mmol H_2_O m^−2^ s^−1^),	−0.77^***^	0.51^***^	0.35^***^		−0.82^***^
Leaf temperature depression (*LTD*, °C)		−0.42^***^	−0.21	−0.77^***^	0.67^***^
Stomatal limitation value (*g_lim_*)	0.67^***^	−0.09	−0.15	−0.82^***^	

When the relationship was linear we used Pearson correlation, otherwise we used Spearman. Genotype mean values were used in the correlation analysis, ^*^, ^**^, and ^***^ represent probability levels of significance of 0.05, 0.01, and 0.001, respectively.

**Figure 2:**
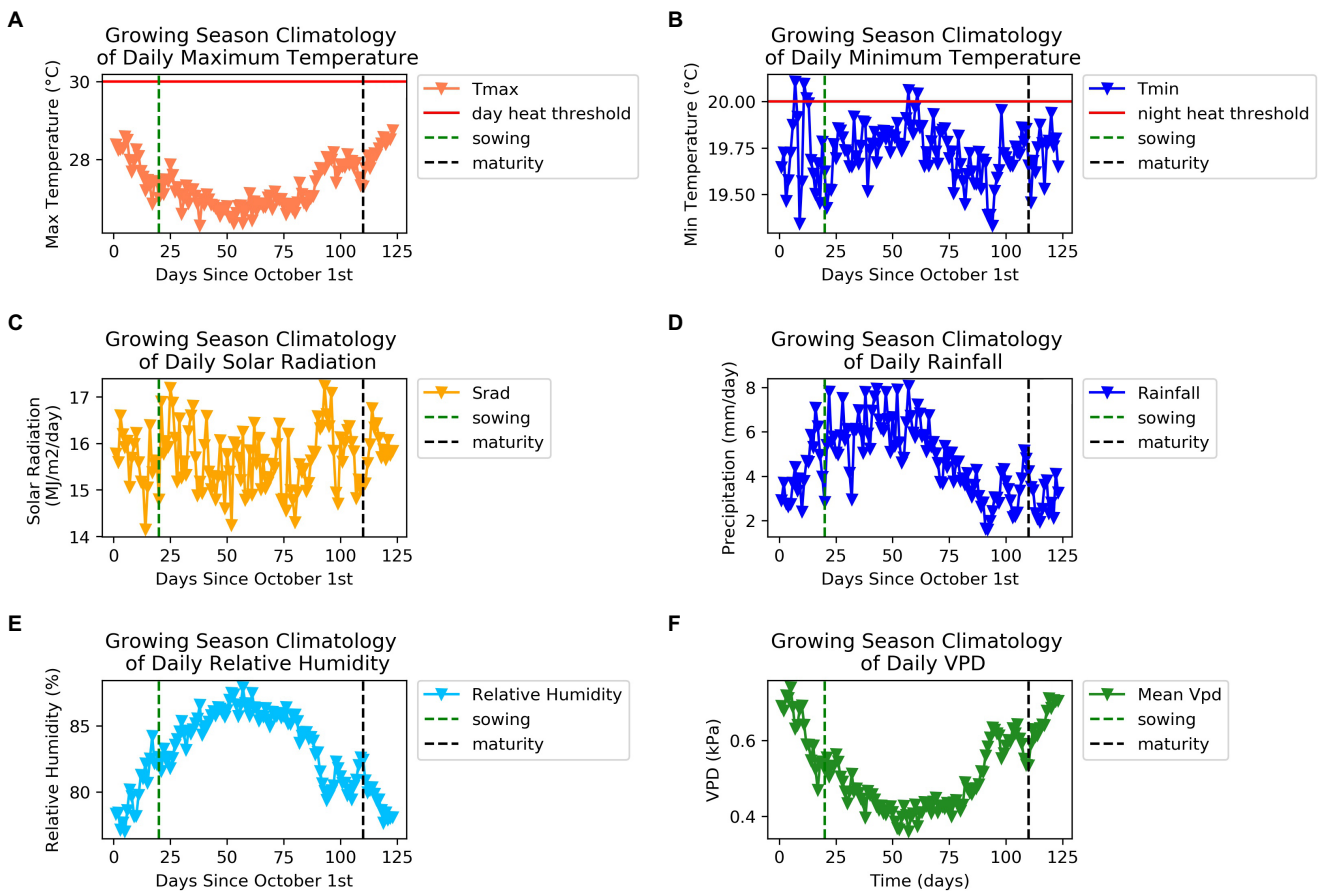
Weather characteristics of the main growing season in Florencia for the years 1984–2014 **(A)** Daily maximum temperature (°C), **(B)** daily minimum temperature (°C), **(C)** daily solar radiation (MJm^−2^ day^−1^), **(D)** daily rainfall (mmday^−1^), **(E)** relative humidity (%), **(F)** daily vapour pressure deficit (kPa).

**Figure 3:**
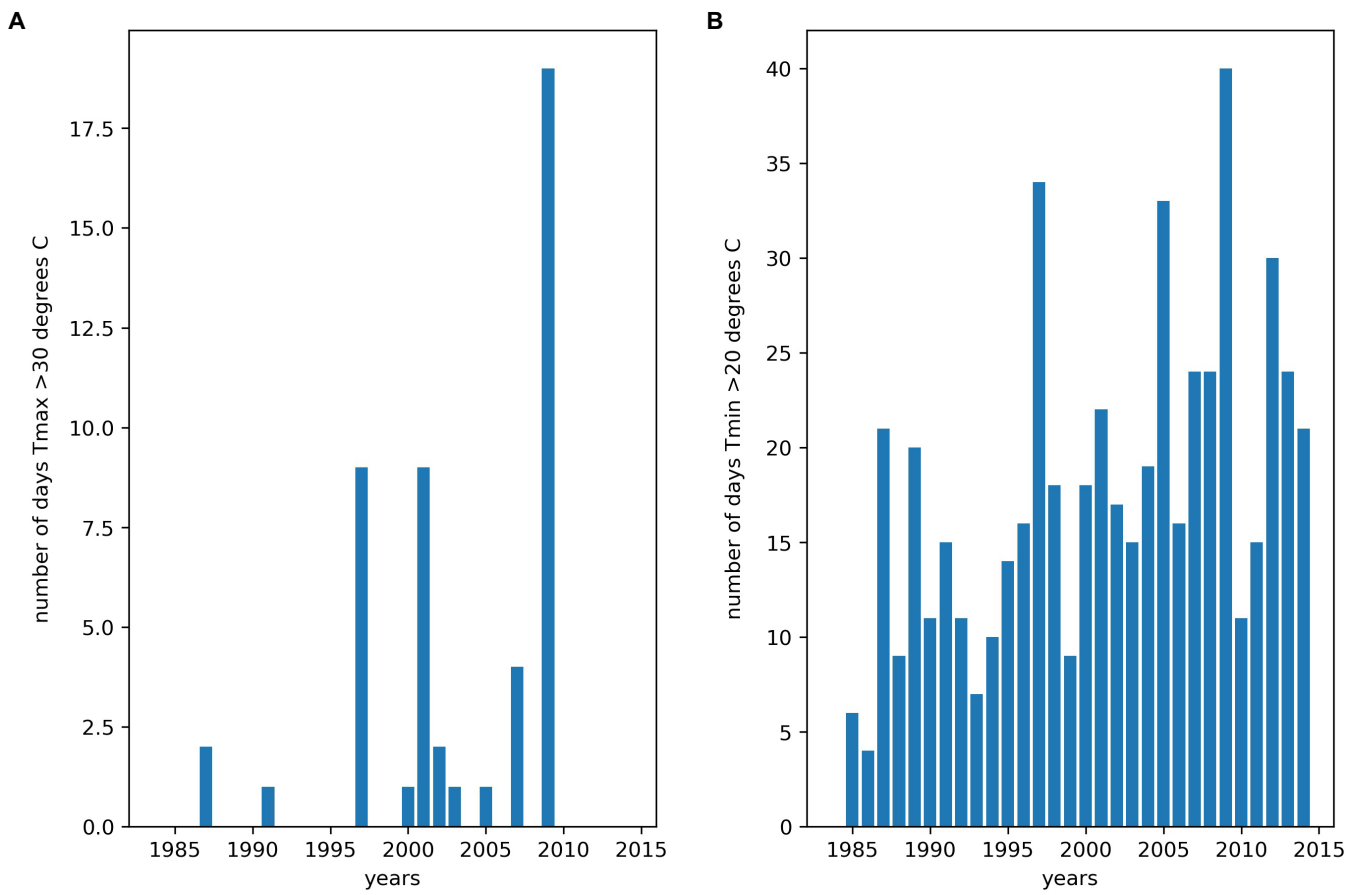
Number of days daytime and nighttime thresholds were exceeded between 20 and 60days into the main growing season for the years 1984–2014 in Florencia. **(A)** day time heat stress thresholds were exceeded, and **(B)** night time heat stress thresholds were exceeded.

We summarized the weather characteristics of the main growing season in Florencia for the years 1984–2014 (Figure 2). The growing season is characterized by higher values of maximum and minimum temperatures and plenty of rainfall in the first half of the season. Relative humidity is extremely high in this tropical rainforest environment and when combined with low wind speeds leads to very low values of *VPD* throughout the season. This suggests weaker atmospheric demand for water than would be the case in most bean growing regions. Average daily maximum temperatures greater than 30°C and average daily minimum temperatures greater than 20°C are thought to reduce bean yields (Porch et al., 2010). Between the years 1984 and 2014, approximately a third of growing seasons experienced at least 1day in which maximum daily temperatures exceeded 30°C between 20 and 60days into the growing season (Figure 3). Average daily minimum temperatures of greater than 20°C were experienced in all growing seasons between 20 and 60days into the growing season. This suggests that high nighttime temperatures are more of a production risk than high daytime temperatures in Florencia.

### Physiological Characteristics Among Common Bean Genotypes When Grown Under High Temperature Conditions

**Figure 4:**
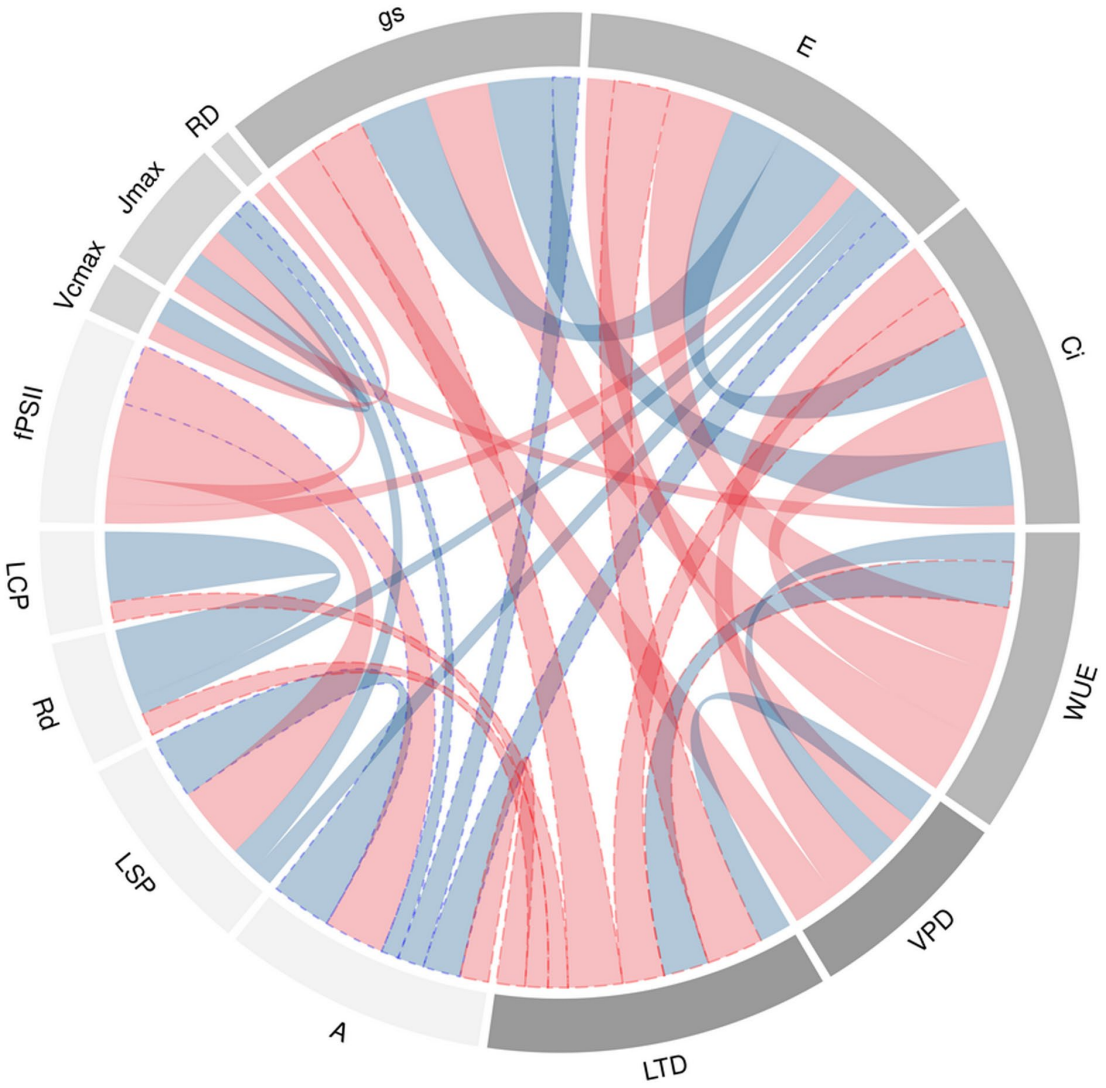
Chord diagram of correlation coefficients between agronomic and physiological traits at leaf level of 64 genotypes of bean grown under conditions of high temperatures. The ribbons within the circle correspond to significant correlations with a *p*<0.05. The red ribbons indicate negative coefficients and the blue ribbons indicate positive coefficients. The ribbons with blue and red dotted lines are the relationship of *A* and *LTD* to the different variables whose correlation was statistically significant. The acronyms are described in Table 1.

Chord diagram of correlation coefficients between agronomic and physiological traits at leaf level of 64 genotypes of bean grown under conditions of high temperatures is presented in Figure 4.

In general, grain yield (*GY*) of the 64 bean genotypes ranged from 336 to 1,614kgha^−1^ with an average value of 957kgha^−1^ (Table 2). The genotypes with the higher values of *GY* were BFS 10, SEN 52, SER 323, SCR 56, SEF 10, SEF 40, SEF 70, SEF 73, SMR 173 and SMN 99 with a production value higher than 1,300kgha^−1^. Within the genotypes that have higher *GY*, differences were found at the *LTD* level (Supplementary Figure 1A). For example, BFS 10, SMN 99 were the genotypes with higher *E*, which translated into a lower *LTD* (more negative), contrary to what was found in the different SEFs lines (SEF 10, SEF 40, SEF 70, SEF 73), SMR 173, SER 323, and SEN 52. Some genotypes found in the lower left quadrant (SER 48, ALB 348, RRA 80) have low *E* and positive *LTD* which negatively affected *A* and *GY*. However, sometimes this mechanism was not efficient enough in the process of grain formation (ALB 121, SEF 14, SEF 12) probably because of additional effects from soil constraints.

**Table 2:** Physiological variables of the different types of common bean genotypes grown at high temperatures.

Variable	EUW				OEUW				WUE				General			value of *p*
	Media		E.E		Media		E.E		Media		E.E		Media		E.E.	
GY	1182.5	±	84.6	a	990.3	±	67.2	a	835.3	±	52.8	b	959.7	±	40.5	0.0057
*A*	24.1	±	1.0	a	22.9	±	1.4	a	20.8	±	1.0	b	22.2	±	0.7	0.0010
*LSP*	965.1	±	65.1		958.1	±	52.6		913.9	±	72.1		938.9	±	39.8	
R_d_	1.0	±	0.1	b	1.8	±	0.1	a	0.6	±	0.1	c	1.1	±	0.1	< 0.0001
LCP	26.3	±	2.8	b	45.6	±	3.0	a	18.2	±	2.3	c	28.5	±	2.1	< 0.0001
*Φ*_PAR_	1.6	±	0.1		1.7	±	0.1		1.8	±	0.1		1.7	±	0.1	
*V*_cmax_	34.0	±	6.6	b	61.5	±	8.2	a	41.7	±	4.6	b	46.2	±	3.8	0.0166
*J*_max_	51.3	±	6.2		60.2	±	5.2		71.7	±	6.5		63.6	±	3.8	
*R*_D_	18.2	±	1.5		15.4	±	1.3		17.3	±	1.0		16.9	±	0.7	
*g_lim_*	0.19	±	0.01	c	0.35	±	0.02	b	0.58	±	0.04	a	0.43	±	0.03	< 0.0001
*gs*	863.1	±	78.3	a	357.4	±	33.2	b	199.6	±	18.9	c	394.1	±	38.9	< 0.0001
*C*_i_	297.4	±	4.6	a	235.5	±	10.0	b	175.4	±	12.7	c	220.9	±	9.0	< 0.0001
*WUE*	2.6	±	0.2	c	3.7	±	0.2	b	5.5	±	0.4	a	4.3	±	0.2	< 0.0001
*E*	9.8	±	0.8	a	6.0	±	0.5	b	4.1	±	0.3	c	5.9	±	0.4	< 0.0001
*LTD*	−1.37	±	0.5	c	−0.04	±	0.2	b	0.81	±	0.2	a	0.07	±	0.2	< 0.0001

*EUW, Effective Use of Water; WUE, Water use efficient; OEUW, Opportunistically effective use of water. The acronyms are described in Table 1. Mean±Standard error. a, b, c: Averages with a letter in common between rows are not significantly different at 5% probability*.

The BFS 10 showed the highest value of *g_s_* and *GY*, but also high *E* that affected *LTD*. Interestingly, the SCR 56 and several SEFs lines (SEF 10, SEF 40, SEF 70, SEF 73) showed opposite behavior characterized by low *gs*, high *g_lim_*, and a high *GY*. We found that for low left quadrant genotypes that have low *g_s_*, the g_lim_ was higher, and therefore, the leaf temperature was higher than the air temperature (positive *LTD*) with negative effect on *GY*. Other genotypes with similarly high *GY* values presented a lower *E* and high *WUE* that caused higher leaf temperature than air temperature (Supplementary Figure 1D).

When analyzing the effect of increased air temperature on carbon assimilation and its effect on water use efficiency, we found that some genotypes had higher *E* and therefore more negative *LTD*, a mechanism that allowed higher *A* (Supplementary Figure 2A). In particular, the SCR 56, SEN 52, and SEF 40 genotypes presented both higher *WUE* and *A*. This higher efficiency is mainly due to the maximum rate of RuBisCO carboxylation (*V*_cmax_) presented specifically for SEF 40 (Supplementary Figure 2B). The physiological performance of ALB 352 and SMR 152 which presented higher *A* with lower *V*_cmax_ and *GY* value higher than 1,100kgha^−1^ due to a low g_lim_ (Supplementary Figure 2C) is worth mentioning. As the air temperature increases, g_lim_ increases, decreasing *C*_i_ and consequently the *A*. In this sense, we described two interesting groups of bean genotypes [(1) SMN 99, SMN 95, SEF 14, ALB 121 and SMR 123, and (2) ALB 352, SMR 174, and SEN 52] which with similar *C*_i_, achieved high carbon assimilation [*A*, in group (1) 22, and group (2) 30μmol CO_2_ m^−2^ s^−1^, respectively] under very different leaf temperatures. These two responses suggest different physiological behavior as mechanisms of leaf-lead air temperature dissipation and reveal a physiological or biochemical player in the background (Supplementary Figure 2C). A reduction of the *Φ*_PAR_ was found related to increase of *LSP* (SCR 56, SEF 70 and SEF 10; Supplementary Figure 2D), which affects the assimilation of carbon (*A*) and a maximum rate of electron transport driving regeneration of ribulose-1, 5-bisphosphate (RuBP; *J*_max_). These genotypes were able to achieve very high *GY* (above 1,450kg) despite leaf temperatures more than +1°C above ambient temperatures (Supplementary Figures 1D and 2D).

**Figure 5:**
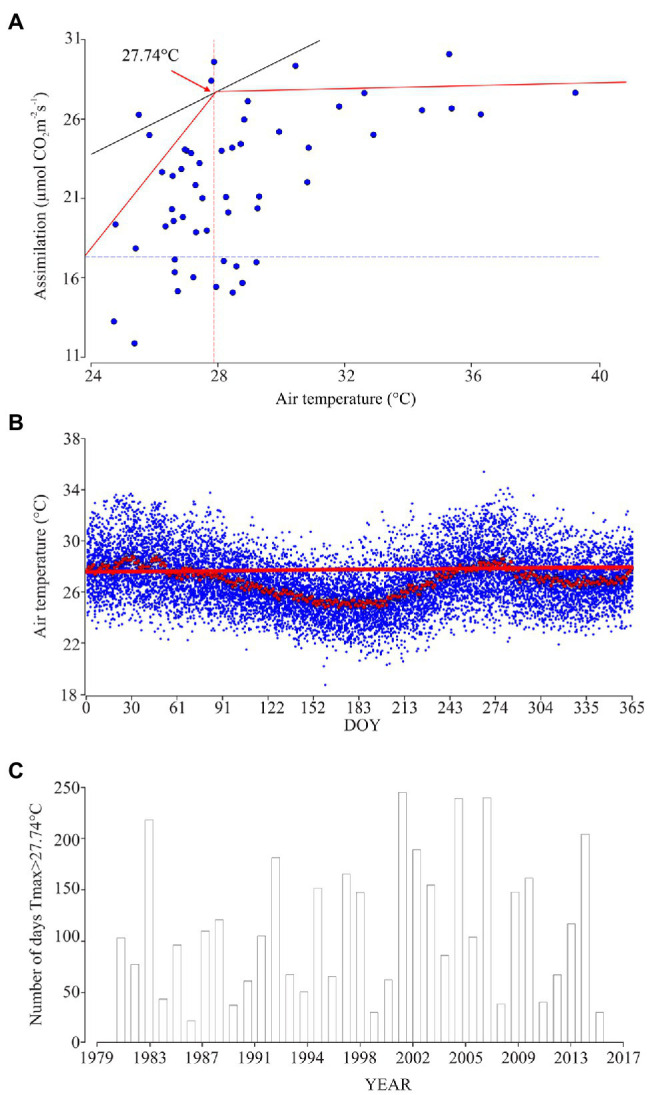
Relationship between physiological behavior and air temperature. **(A)** Linear segments model between ambient temperature and A: Red line is the path of the adjusted linear segment model, of the value 27.74°C, which corresponds to γ (the point at which the segments join). This point allows one to determine the maximum temperature threshold at which the bean under normal conditions performs the carbon fixation process effectively without enzymatic or morphological restrictions. **(B)** Daily temperature behavior between 1979 and 2017 (blue points); red points are the averages and standard error for each day (*n*=38years). Red line corresponds to γ at which the segments join 27.74°C. **(C)** Number of days in each year with maximum temperature values above 27.74°C.

Two-segment model between the ambient temperature and *A* showed the point at which the segments join. In our study, this point was found at 27.74°C (Figure 5A). This point reveals the air temperature at which *A* reaches its maximum and then stays linear, with a slope of 0.63 (*p*<0.001). From the historical climate information, we found 85days of the year that have values higher than 27.74°C, distributed in two periods of the year that correspond to Julian days 1 to 53 (between the months of January to February) and Julian days 243 to 284 (that correspond to the whole month of October and first week of November; Figures 5B,C).

### Water Use as a Mechanism to Tolerate High Temperatures

**Figure 6:**
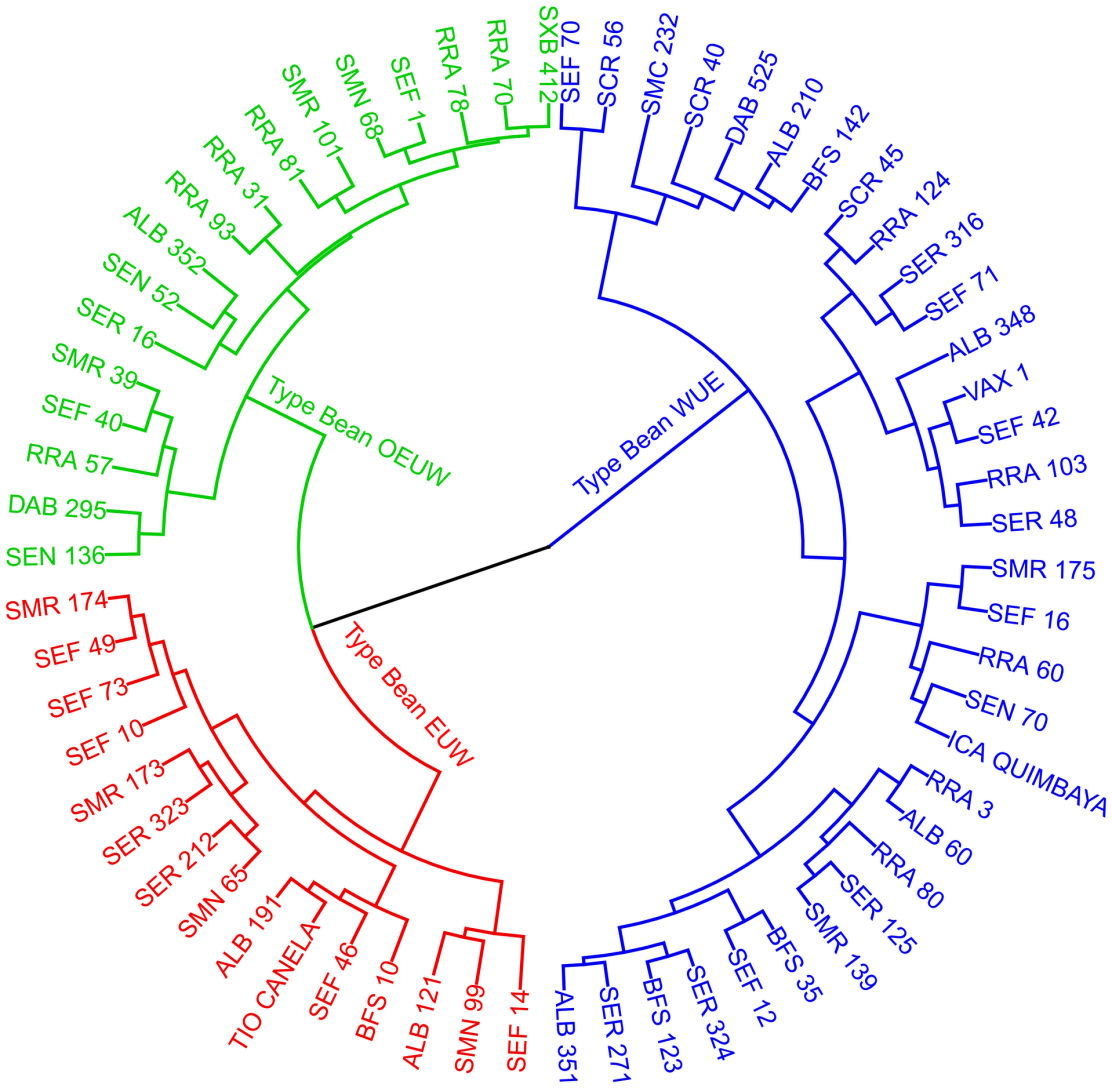
Dendrogram (method Ward, Euclidean distance) showing the different typologies of common bean genotypes grouped by different physiological variables (Table 1) cultivated at high temperatures. EUW; Effective Use of Water, WUE; Water use efficient, OEUW; Opportunistically effective use of water.

According to the cluster analysis of different variables taken from the 64 bean genotypes, three statistically different groups with contrasting physiological mechanisms to tolerate high air temperature were found. According to their physiological traits, we divided the bean genotypes into: (1). *WUE*: Water Use Efficiency, (2). *EUW*: Effective use of water, and (3). *OEUW*: Opportunistically effective use of water (Figure 6). These typologies were generated by trait differences (*p*<0.0001) using leaf temperature differential (*LTD*), instantaneous water use (*WUE*), the transpiration (E), gas exchange (*g*_s_), gasometric limitation caused by air temperature increase (g_lim_), and carbon use and fixation (*C*_i_, *R*_d_, *LCP* and *V*_cmax_).

The PCA (Supplementary Figure 3A) related to gas exchange (*g*_s_, g_lim_), water use (*WUE*, *E*), intracellular carbon (*C*_i_) and leaf temperature differential (*LTD*) is clearly opposed along axis 1 (32.8% of variance can be explained), separating genotypes with contrasting differences in heat dissipation strategy and water use. Axis 2 (20.5% variance can be explained) opposed genotypes with characteristics related to *Φ*_PAR_ and *LSP*. The separation of bean genotype into three typologies according to physiological traits was significant and explained 30% of the total variance according to the Monte Carlo test (Supplementary Figure 3B).

Below we describe the representative typologies. We take only those genotypes with a GY higher than 1,200kg to eliminate genotypes with very low yield potential or heavily damaged ones. The main differences are due to most of the variables considered in this study. However, *LSP*, *Φ*_PAR_, *J*_max_ and *R*_D_ did not show statistical differences in the typologies (Table 2).

#### Genotypes With *EUW* (*n*=14; 21.8% of the Total Genotypes Evaluated)

**Figure 7:**
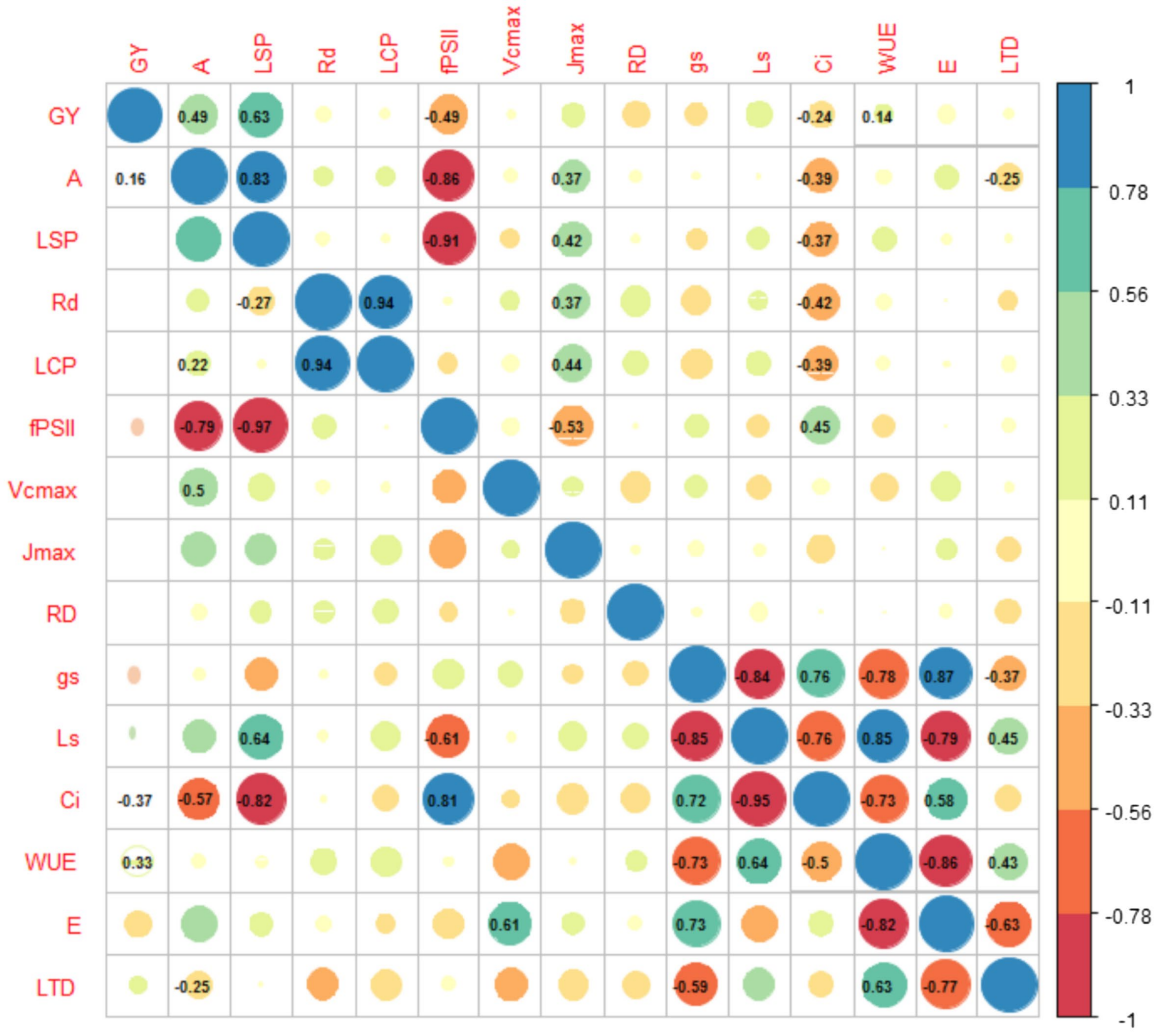
Correlation between the different physiological variables of different genotypes grown at high temperatures. Below and above the diagonal, values correspond to the correlations for the EUW (Effective Use of Water) and WUE (Water use efficient), respectively. The blue and red are the positive and negative correlation, respectively, accompanied by the magnitude of the correlation by the size of the circle. Only significant correlations (*p*<0.05) appear within the circles. The acronyms are shown in Table 1.

This group on average has a grain yield of 1,182kg, represented by genotypes such as BFS 10, SER 323, SMN 99 (*Phaseolus vulgaris*) and SEF 10, SEF 46 and SEF 73 (*P. vulgaris*, *P. acutifolius* and *P. coccineus interspecific*). The two important mechanisms involved (transpiration and thermal dissipation by *LTD*) reduced g_lim_, increasing *g_s_* and *C*_i_, and therefore carbon fixation (*A*, Table 2), resulting in high *GY*. The negative *LTD* values (i.e., the leaf is cooler than the air temperature), should be involved as a heat resistant genotype trait (similar trend is observed in second typology, *WUE;* Deva et al., 2020). Not surprisingly, when analyzing the correlations between the different variables for this typology, we found that gs exhibited high values in this group (Figure 7). Taken together, the high *GY*, with high *g*_s_ and E, and consequently low leaf temperature, allows categorization of this group as heat resistant genotypes with profligate water spenders traits (similarly to Blum, 2009).

#### Water Use Efficient Genotypes (*n*=30; 46,8%)

This group is represented by genotypes such as SEF 70 (*P. vulgaris*, *P. acutifolius*, and *P. coccineus interspecific*) and SCR 56 (*Phaseolus vulgaris*) with *GY* of 1,493 and 1,479kgha^−1^, respectively. The average *GY* for this typology is 835kgha^−1^. This group represented the highest values of *WUE*, hence its name. This high efficiency had a notable impact on the capacity to carry out gas exchange processes, reducing *C*_i_ and *E*, which increased g_lim_. In turn, the decrease in *E* was followed by increased LTD (Leaf temperatures were sometimes higher than air temperatures). Despite this, SEF 70 and SCR 56 showed higher capacity of translocation of photoassimilates (enhanced seed filling) likely because of their high *LSP* which translated into an increase in the maximum rate of electron transport driving regeneration of ribulose-1, 5-bisphosphate (RuBP; *J*_max_). This resulted in an increase in the maximum rate of RuBisCO carboxylation (*V*_cmax_). The efficiency of the PSII is also demonstrated as it was able to regulate the excess energy and to carry it along the electron transport chain. The correlations of this typology were largely related to those variables related to carbon use (*A*, *R*_d_, *LCP*), as well as reduced *C*_i_, since this typology has a high g_lim_ (Figure 7).

This group demonstrates typical conservative traits of the water savers group (high *WUE*, positive *LTD*, high g_lim_, low *E*, *g_s_*, *C*_i_ etc.). Generally, in some terminal drought scenarios, these traits can increase final *GY*. However, the lowest *A*, *R*_d_ and *LCP* which altogether surely lead to lowest *GY* proved that conservative crop behavior can significantly decrease the yield potential upon heat in Western Amazonia (in agreement with hypotheses postulated in Deva et al., 2020). However, our recommendations are that water savers should be selected only if they reach above-average yield and should not be automatically neglected in breeding programs for increasing resistance to heat, especially for their water-saving tendencies. In our study, some genotypes from this group showed different pathways to heat resistance. Although the real crop water consumption was not measured in this study and the field was irrigated up to field capacity, based on heat-influenced gasometric values (*A, g_s_*) we can postulate this group as conservative in water use despite water availability.

#### Genotypes With *OEUW* (*n*=20; 31,2%)

This group of bean genotypes had an average *GY* of 990kgha^−1^, which was statistically higher than the *WUE* typology. The group is characterized by genotypes such as SEN 52, SMR 173, and SMR 39 (*Phaseolus vulgaris*) with yields of 1,608, 1,346 and 1,205kgha^−1^, respectively, as well as SEF 40 (*P. vulgaris*, *P. acutifolius* and *P. coccineus*) with a yield of 1,557kgha^−1^. The main characteristic of this typology is related to higher values of *R*_d_ and *LCP* and efficiency in carbon fixation (*V*_cmax_). At the same time, with its medium capacity in the instantaneous photosynthetic use of water (*WUE*), middle *E*, middle *C*_i_, g_lim_ and *g*_s_, these genotypes regulated leaf temperature less effectively than EUW group. However, this finely regulated water usage allowed efficient carbon fixation, achieving statistically similar performance that achieved heat-resistant ones through transpiration cooling (*EUW* typology). SER 16 was previously characterized as a typical high-yielding water saver in terminal drought. However, in our study, Western Amazonian soil allowed only very low yield performance of SER 16. We can characterize this third group *OEUW* as heat resistant genotypes with opportunistic water spender strategy and photosynthetic heat acclimation (Wang et al., 2020) with hybrid trait combinations of both above-mentioned typologies.

**Figure 8:**
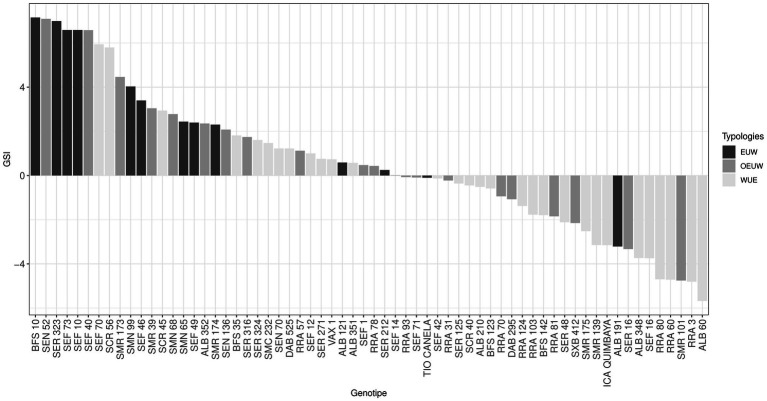
Genotype stress index of common bean genotypes cultivated at high temperatures. EUW: Effective Use of Water, WUE: Water use efficient, OEUW: Opportunistically effective use of water.

By analyzing heat stress resistance in beans under the western Amazonian conditions using the genotype stress index (*GSI*) we found differences between genotypes (Figure 8). Genotypes below the general average were mainly from the *WUE* typology group. Conditions in the Amazonia make them susceptible to heat and acidic soil resulting in reduced grain yield. However, genotypes such as BFS 10, SEN 52, SER 323, different SEFs (SEF 73, SEF 10, SEF 40, SEF 70), SCR 56, SMR 173 and SMN 99 (mainly from *EUW* group) showed *GSI* values higher than three due to their ability to adapt.

## Discussion

### Heat Tolerance Mechanisms

It has been shown that high temperature impacts phenology and grain yield in Western Amazonia (Suárez et al., 2018a, 2020). In the present study, we identified physiological heat tolerance mechanisms related to gasometric water use. We identified how some common bean lines maintain efficiency in mobilizing photosynthates for grain formation under high temperature conditions. Some lines such as BFS 10, SEF 10, SEF 73, SER 323, SMN 99 and SMR 177 which can be classified as anisohydric (water-spending) genotypes, use the mechanism of leaf temperature differential (*LTD*, Habermann et al., 2019; Deva et al., 2020). This trait is associated with increased gas exchange (*g_s_*), i.e., an increase of the substrate (*C*_i_) for carbon fixation (*A*) at the cellular level by increasing also *E*. Therefore, when *E* increased, some bean genotypes—through latent heat transfer—maintained a lower leaf temperature relative to that of the air (Traub et al., 2018). Therefore, transpiration cooling allowed these genotypes to maintain the photosynthetic processes without major impacts from high temperatures, which was supported by the positive correlation obtained in our study between *LSP* with *A* and *GY*. To work this mechanism properly (*LTD*), water must be exchanged for carbon and carbon then incorporated into biomass, which relates to effective use of water (*EUW*; Salvucci and Crafts-Brandner, 2004; Blum, 2009; Boomiraj et al., 2010; Hall, 2010; Urban et al., 2017; Dubey et al., 2020). If this mechanism does not operate some genotypes may have stomatal limitations (g_lim_), reducing latent heat flow, thus increasing leaf temperature (Orozco et al., 2012). On the other hand, isohydric (“water-saving”) genotypes such as SEF 70 and SCR 56 presented low *E* that caused a greater increase in *LTD*. These genotypes were characterized by having the ability to have greater *WUE*, which is attributed to higher water content within the leaves, which allowed leaf-morphology-related thermal capacity to sustain *gs* under high temperatures (Blum, 2009; Albrecht et al., 2020; Eustis et al., 2020). However, water savers in drought conditions are known to have low biomass accumulation and likely lower yield in comparison with spenders (dependent on water-availability scenario, if early, intermittent or terminal drought occur). Other physiological characteristics presented by this type of bean genotype was high maximum photochemical efficiency of PSII, due to its high *LSP* that regulated excess energy along the electron transport chain (Robledo et al., 2010; Pan et al., 2018) and to influence a higher maximum regeneration rate of ribulose-1,5-bisphosphate (RuBP; *J*_max_). Another group made up of SEF 40 and SEN 52 which, in addition to having very similar characteristics to the *WUE* typology (anisohydric) presented a higher maximum carboxylation rate (*V*_cmax_), and higher *R*_d_ and *LCP* compared to the other typologies. Interestingly, in some cases the rate of photosynthesis was not limited by the ability of the enzyme Ribulose-1,5-bisphosphate (RuBP) carboxylase/oxygenase to carboxylate RuBP (*V*_cmax_) since the electron transport rate for the regeneration of RuBP was also not affected. The mechanism of this typology is to save soil water, dissipate to some degree the negative effect of air temperature but still remain efficient in carbon fixation and assimilate translocation leading to higher grain yield. It has been recently mentioned that a thermal acclimatization has allowed a coupling between *V*_cmax_ and *R*_d_ (Wang et al., 2020). Therefore, the third typology *OEUW*, besides having a high *V*_cmax_, also its *R*_d_ and *LCP* values were higher in comparison with the other typologies. The increase in temperature in these bean genotypes could lead to a higher demand for maintenance which is related to higher *R*_d_ values (Smith et al., 2019).

### Genotypic Variation in Mechanisms of Heat Tolerance

According to the mechanisms described by the different typologies of beans, we found that some of them related to the use of water and others to the ability to increase carboxylation rate. We found that in each typology there were genotypic variations that allowed grain yields over 1,200kgha^−1^. These genotypes were called advanced superior lines with better heat resistance-related traits for better physiological performance. For example, BFS 10 and SER 323 were genotypes that were grouped into a single typology (*EUW*) with increased *E* generating an efficient canopy cooling (Hong et al., 2003). High *E* was translated into a more negative LTD––a situation that generated conditions within the canopy to maintain physiological processes. This mechanism (better canopy microclimate) hypothetically allowed a translocation of photosynthates for grain formation, thus increasing the *GY*. Behavior of two genotypes with highest GSI (BFS 10, SER 323; both *EUW* group) showed the pragmatic base for the above-mentioned statement: although they both had negative *LTD*s differences were found in carbon fixation efficiency that allowed high *GY* performance for both genotypes. However, the connection between *E*, *g*_s_, and overall water uptake and *GY* need to be further studied.

A genotype that showed optimal performance under high temperatures in terms of mobilization of photosynthates for grain formation was SEN 52. This is a black-seeded line derived from the cross [(SXB 123×DOR 677)×SEN 34], which has been reported as heat resistant (CIAT, 2015). This genotype for its high performance can be used as parental material to develop common bean lines tolerant to high temperatures. It was found that this genotype used a certain amount of water to allow an increase in the stomatal conductance (it belongs to *OEUW* typology). Generally, under hot conditions the limitation of *A* is presented by a decrease in both the maximum rate of carboxylation (*V*_cmax_), the maximum rate of RuBP regeneration (*J*_max_) and the maximum photochemical efficiency of PSII (Yamori et al., 2011; Haque et al., 2017). However, SEN 52 did not show negative heat effects on diffusion and assimilation of carbon. Taken together, the most important strategy used by SEN 52 is to use a mechanism that combines *EUW*, *LTD*, light use (*Φ*_PAR_) and higher carbon fixation (Huang et al., 2017).

Other bean genotypes that showed heat resistance traits were SEFs (SEF 10, SEF 40, SEF 70 y SEF 73) that were generated by crossing (ALB 74×INB 841) F_1_×RCB 593, where ALB 74 provides genes from *P. coccineus* L. and INB 841 provides genes from *P. acutifolius* A. Gray. This allowed them to obtain genetic gain by inheriting its heat resistance (Polanía et al., 2017). When we analyzed in detail the gas exchange mechanisms, we found for example, SEF 40 (the *OEUW* type) was more efficient in fixing carbon (*V*_cmax_) and showed highest photosynthetic values observed in this study. SEF 70 was the most water efficient genotype compared to the others, with a high light saturation point (*LSP*) where the functionality of the PSII in relation to electron transfer was efficient. A common feature of SEF 10 and SEF 73 was high instantaneous water use.

In this study, we elucidated the physiological mechanisms of ten heat-resistant genotypes (based on *GY* upon heat: BFS 10, SCR 56, SEF 10, SEF 40, SEF 70, SEF 73, SEN 52, SER 323, SMN 99 and SMR 177). The increase in temperature affected the adjustment of the thermal tolerance of PSII (Crafts-Brandner and Salvucci, 2002) due to possible reassembly of the reaction centers during heat stress (Lu et al., 2017). We found that PSII was sensitive to high temperatures (Camejo et al., 2005; Feng et al., 2014; Chen et al., 2017) presenting effects on the maximum Rubisco carboxylation rate (Chen et al., 2005; Haque et al., 2017) According to Blum (2009) under water and high temperature stress a high continuous stomatal conductance is required, to allow greater CO_2_ fixation that translates into a maximum use of soil water for transpiration (which is expressed actually by a lower *WUE)*. However, we found another successful mechanism that some genotypes besides having a moderate use of water have developed a high carbon fixation efficiency (described for the genotype SEN 52).

**Figure 9:**
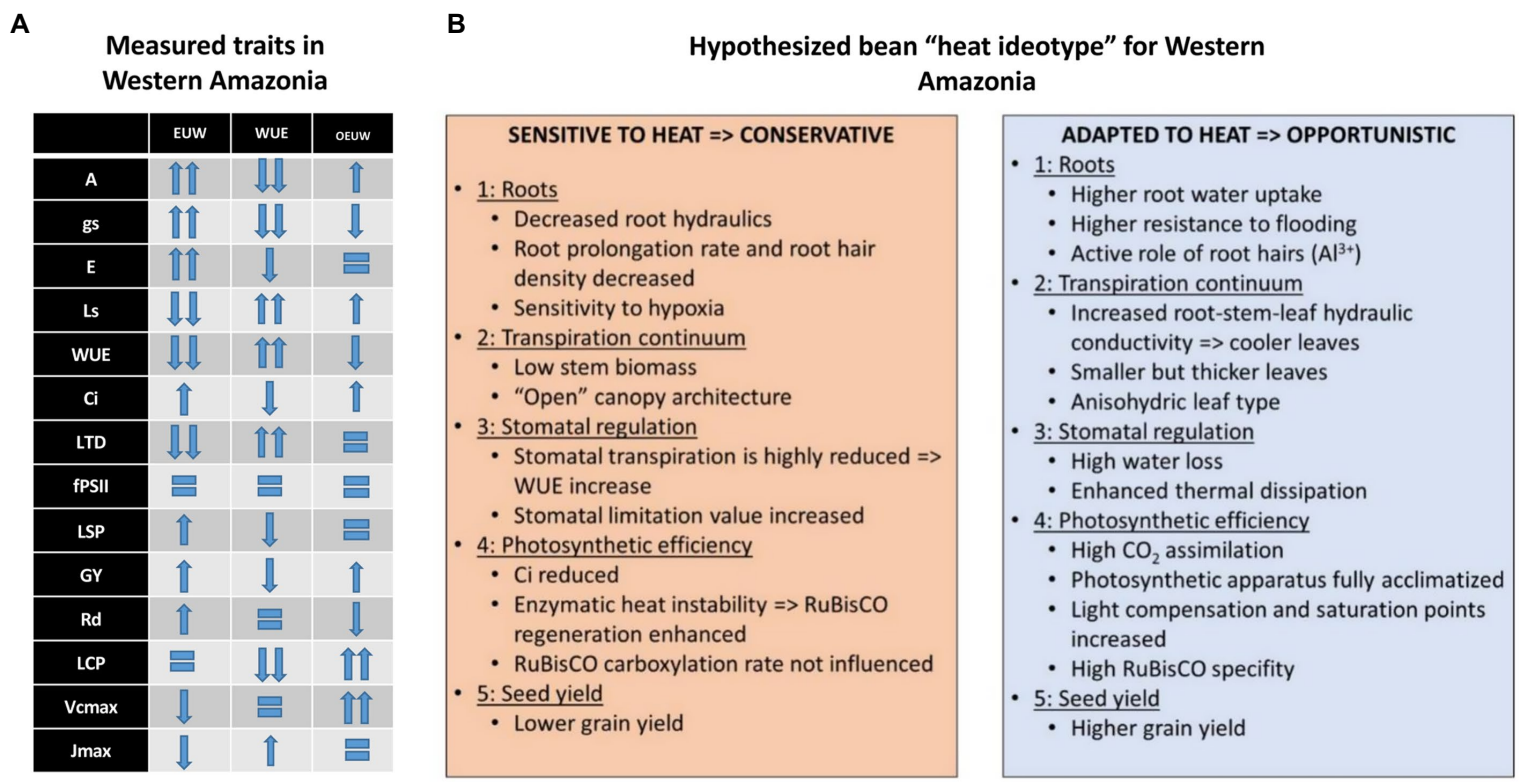
The overview of the measured physiological traits **(A)** in heat-treated beans in Western Amazonia for three different genotype groups and suggested bean heat ideotype (adapted to heat) based on published and hypothesized results **(B)**. Gas exchange parameters at leaf level: stomatal conductance (*gs*, mmol H_2_O m^−2^ s^−1^), transpiration rate (*E*, mmol H_2_O m^−2^ s^−1^), photosynthetic Water Use Efficiency (*WUE*, mmol CO_2_ mol^−1^ H_2_O), sub-stomatal CO_2_ concentration (*C*_i_, μmol mol^−1^), stomata limitation value (*g_lim_*), leaf temperature differential (*LTD*), light-saturated maximum net carbon assimilation rate (*A*_max_, μmol CO_2_ m^−2^ s^−1^), light compensation point (*LCP*, μmol m^−2^ s^−1^), dark respiration rates (*R*_d_, μmol CO_2_ m^−2^ s^−1^), light saturation point (*LSP*, μmol m^−2^ s^−1^), and apparent quantum efficiency (Φ_PAR_, μmol CO_2_ μmol protons^−1^), maximum rate of ribulose-1,5-bisphosphate carboxylase/oxygenase (RuBisCO), carboxylation (*V*_cmax_), maximum rate of electron transport driving regeneration of ribulose-1, 5-bisphosphate (RuBP; *J*_max_), leaf respiration under light conditions (*R*_D_), and yield (kgha^−1^).

In this regard, under the different mechanisms found for heat tolerance, the superior performance of the genotype BFS 10 has been due to its combined resistance to drought (Polania et al., 2016b), as well as tolerance to acid soils and high temperatures (Suárez et al., 2018b, 2020). SMN 99 (heat tolerant; Suárez et al., 2020) and SER 323 performed well under the combined stress of heat and acid soil. Our results showed a *EUW* as a mechanism for heat tolerance, allowing better transpiration cooling associated with final yield (Omae et al., 2012) as an important chronic heat-associated adaptation in common beans. Because the soil water content did not decrease in both experiments and yield differences (visible in absolute yields, yield reductions and stress indices) are consistent between genotypes, we conclude that physiological traits supporting heat tolerance played a similar role and were stable in both experiments. In order to understand differences between the three identified groups we can hypothesize which traits played a bigger role in reducing the negative effect of high temperature during the experiment. The summary of those traits (partially based on measurements, partially based on hypothesized traits) are presented in Figure 9.

## Conclusion

Our climatological analysis (1984–2014) showed that approximately a third of growing seasons experienced at least 1day in which maximum daily temperatures exceeded 30°C and average daily minimum temperatures of greater than 20°C were experienced in all growing seasons. This suggests that high nighttime temperatures are more of a production risk than high daytime temperatures in Florencia of western Amazonia region. Furthermore, climatological profiling for the years 1984–2014 showed that the biggest challenges of growing beans in Western Amazonia are high maximum and minimum temperatures, high rainfall in the first half of the season, high *RH* and low *VPD*.

By analyzing the effect of air temperature on carbon assimilation we found that some genotypes had higher *E* and therefore more negative *LTD*, a mechanism that allowed higher *A* due to the maximum rate of RuBisCO carboxylation (*V*_cmax_) and high rate of electron transport driving regeneration of ribulose-1, 5-bisphosphate (RuBP; *J*_max_). As the air temperature increases, g_lim_ increases, decreasing *C*_i_ and consequently *A*. According to the analysis of different variables taken from the 64 bean genotypes, three statistically different groups were found with contrasting physiological mechanisms: (1) *WUE*: Water Use Efficiency, (2) *EUW*: Effective use of water, and (3). *OEUW*: Opportunistically effective use of water. However, we also found outliers from this rule showing photosynthetically effective genotypes, surprisingly on the background of positive LTD.

Heat-resistant genotypes with effective use of water (EUW) showed profligate spender traits involving the transpiration and thermal dissipation by *LTD with* reduced g_lim_ increasing *g_s_* and *C*_i_ as the most important traits therefore resulting in high *GY*. Heat sensitive water use efficient genotypes (WUE; conservative) represent the highest values of *WUE* with a notable impact on the capacity to carry out gas exchange processes, reducing *C*_i_ and *E*, which increased g_lim_ and reduced LTD. The value of conservative genotypes need to be verified, especially in regions where terminal drought and high temperatures occur together.

By analyzing heat stress resistance in beans under western Amazonian soil and climatic conditions we found that conditions in the Amazonia make beans susceptible to heat and acidic soil resulting in reduced grain yield. However, genotypes such BFS 10, SEN 52, SER 323, different SEFs (SEF 73, SEF 10, SEF 40, SEF 70), SCR 56, SMR 173, and SMN 99 presented high *GSI* values and these genotypes could be suitable for use as parental lines for improving dry seed production.

Further study is needed to verify if mentioned trait combinations are stable over similar environments and quantify their effect on grain yield in heat areas, as water conservative behavior is not expected to increase bean heat resistance in general. Research focused on lysimetric-related water usage under heat stress (i.e., transpiration efficiency, root hairs, root-stem-leaf conductivity accompanied by seed nutritional profiling) of the above-mentioned three groups is recommended to verify the hypothesis that profligate water-spender and not water conservative traits (high *WUE*) can crystallize into higher grain yield in the extreme weather and soil conditions of Western Amazonia.

## Data Availability Statement

The original contributions presented in the study are included in the article/Supplementary Material, further inquiries can be directed to the corresponding author.

## Author Contributions

JS: conceptualization, data curation, formal analysis, funding acquisition, investigation, methodology, project administration, resources, software, validation, visualization, writing – original draft preparation, and writing – review and editing. MU: conceptualization, data curation, investigation, methodology, validation, visualization, writing – original draft preparation, and writing – review and editing. AC: data curation, investigation, methodology, validation, and writing – original draft preparation. JN: data curation, formal analysis, investigation, methodology, and validation. CD: conceptualization, data curation, formal analysis, software, visualization, and writing – review and editing. SB: funding acquisition, resources, validation, and visualization. JP: conceptualization, investigation, methodology, and review and editing. FC: data curation, formal analysis, software, visualization, and writing – review and editing. IR: conceptualization, investigation, methodology, validation, visualization, writing – original draft preparation, and writing – review and editing. All authors contributed to the article and approved the submitted version.

## Funding

CD and MU were supported by a Biotechnology and Biological Sciences Research Council (BBSRC) funded project named Bean Breeding for Adaptation to a Changing Climate and Post-Conflict Colombia (BBACO). Grant number BB/S018964/1.

## Conflict of Interest

The authors declare that the research was conducted in the absence of any commercial or financial relationships that could be construed as a potential conflict of interest.

## Publisher’s Note

All claims expressed in this article are solely those of the authors and do not necessarily represent those of their affiliated organizations, or those of the publisher, the editors and the reviewers. Any product that may be evaluated in this article, or claim that may be made by its manufacturer, is not guaranteed or endorsed by the publisher.
