# Supplementary material for: Water Use, Leaf Cooling and Carbon Assimilation Efficiency of Heat Resistant Common Beans Evaluated in Western Amazonia
Source: Front Plant Sci. 2021 Nov 29;12:644010. doi: 10.3389/fpls.2021.644010 (PMC8667034; doi:10.3389/fpls.2021.644010)
Supplement: Supplementary file 1 [file Data_Sheet_1.docx]

Supplementary Material

# Supplementary Figures and Tables

**
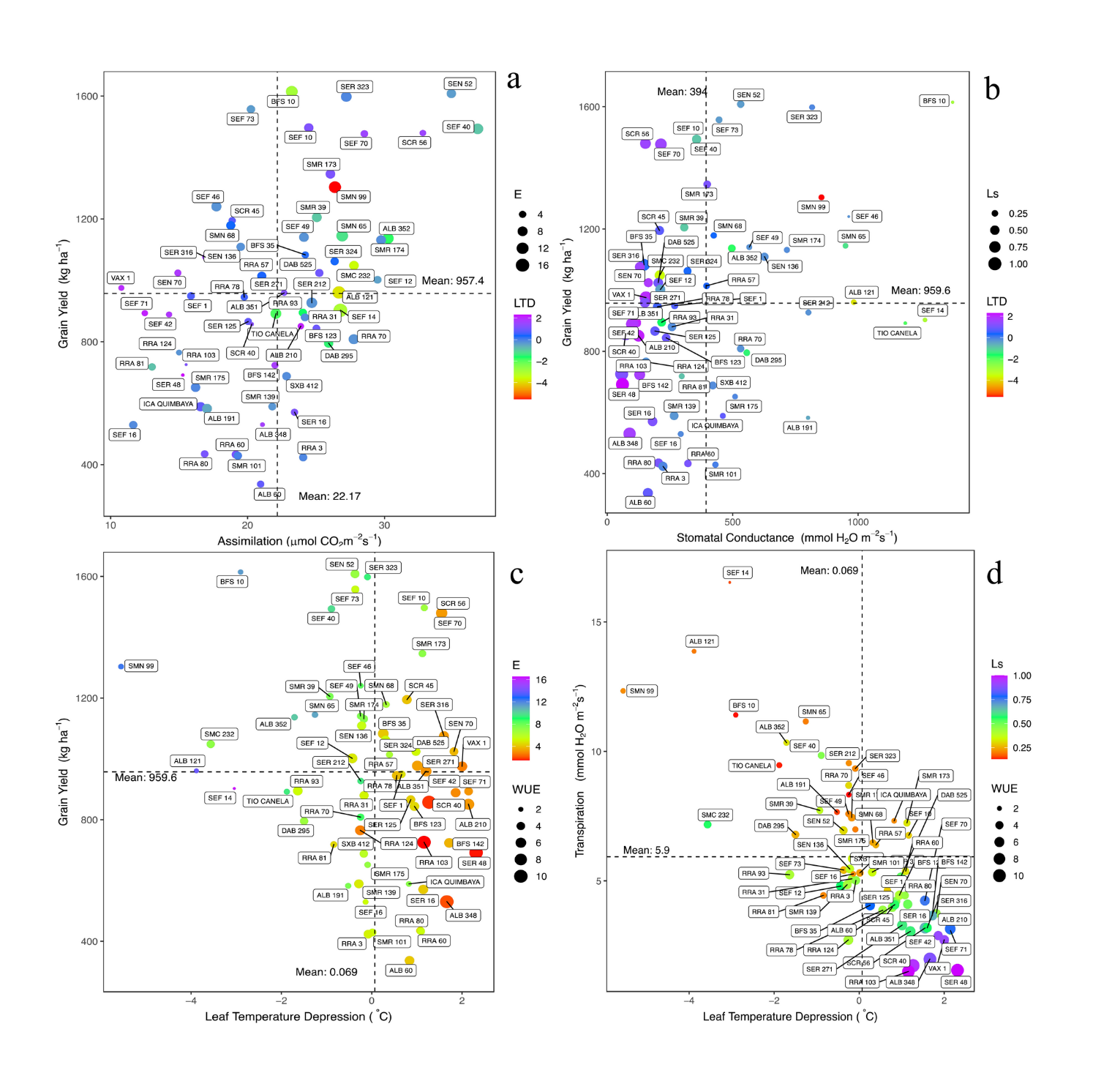
**

**Supplementary Figure 1.** Relationship between different physiological parameters as a function of the gradient (change in the colour of violet and red as greater to lesser value) and magnitude (size of the circle) of parameters that affect the response variable. (A): Grain Yield (*GY*) and carbon assimilation (*A*) as a function of the leaf temperature depression (*LTD*) gradient and the magnitude of Transpiration rate (*E*); (B): Grain Yield (*GY*) and stomatal conductance (g_s_) as a function of the *LTD* gradient and the magnitude of stomatal limitation value (*L*_s_); (C): Grain Yield (*GY*) and *LTD* as a function of the E gradient and the magnitude of photosynthetic Water Use Efficiency (*WUE*); (D): Transpiration rate (*E*) and *LTD* as a function of the *L*_s_ gradient and the magnitude of *WUE*.

**
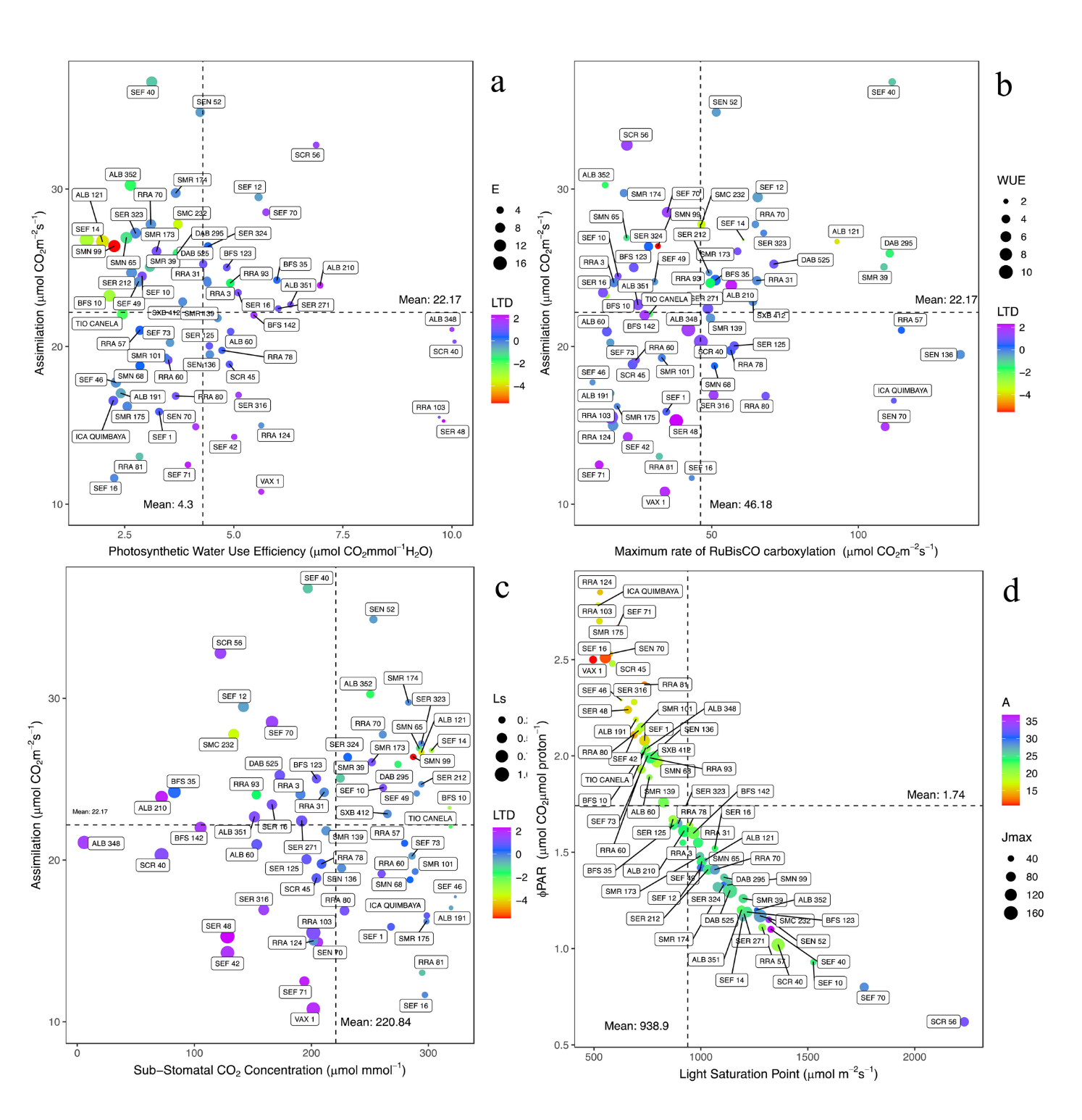
**

**Supplementary Figure 2.** Relationship between different physiological parameters as a function of the gradient (change in the color of violet and red as greater to lesser value) and magnitude (size of the circle) of parameters that affect the response variable. (A): carbon assimilation (*A*) and photosynthetic Water Use Efficiency (*WUE*) as a function of the leaf temperature depression (*LTD*) gradient and the magnitude of Transpiration rate (*E*); (B): carbon assimilation (*A*) and Maximum rate (RuBisCO) carboxylation (*V*_cmax_) as a function of the *LTD* gradient and the magnitude of photosynthetic Water Use Efficiency (*WUE*); (C): carbon assimilation (*A*) and sub-stomatal CO_2_ concentration (*C*_i_) as a function of the *LTD* gradient and the magnitude of stomatal limitation value (*L*_s_); (D): apparent quantum efficiency (*Φ*_PAR_) and light saturation point (*LSP*) as a function of carbon assimilation (*A*) gradient and the maximum rate of electron transport driving regeneration of ribulose-1, 5-bisphosphate (RuBP; *J*_max_).

**
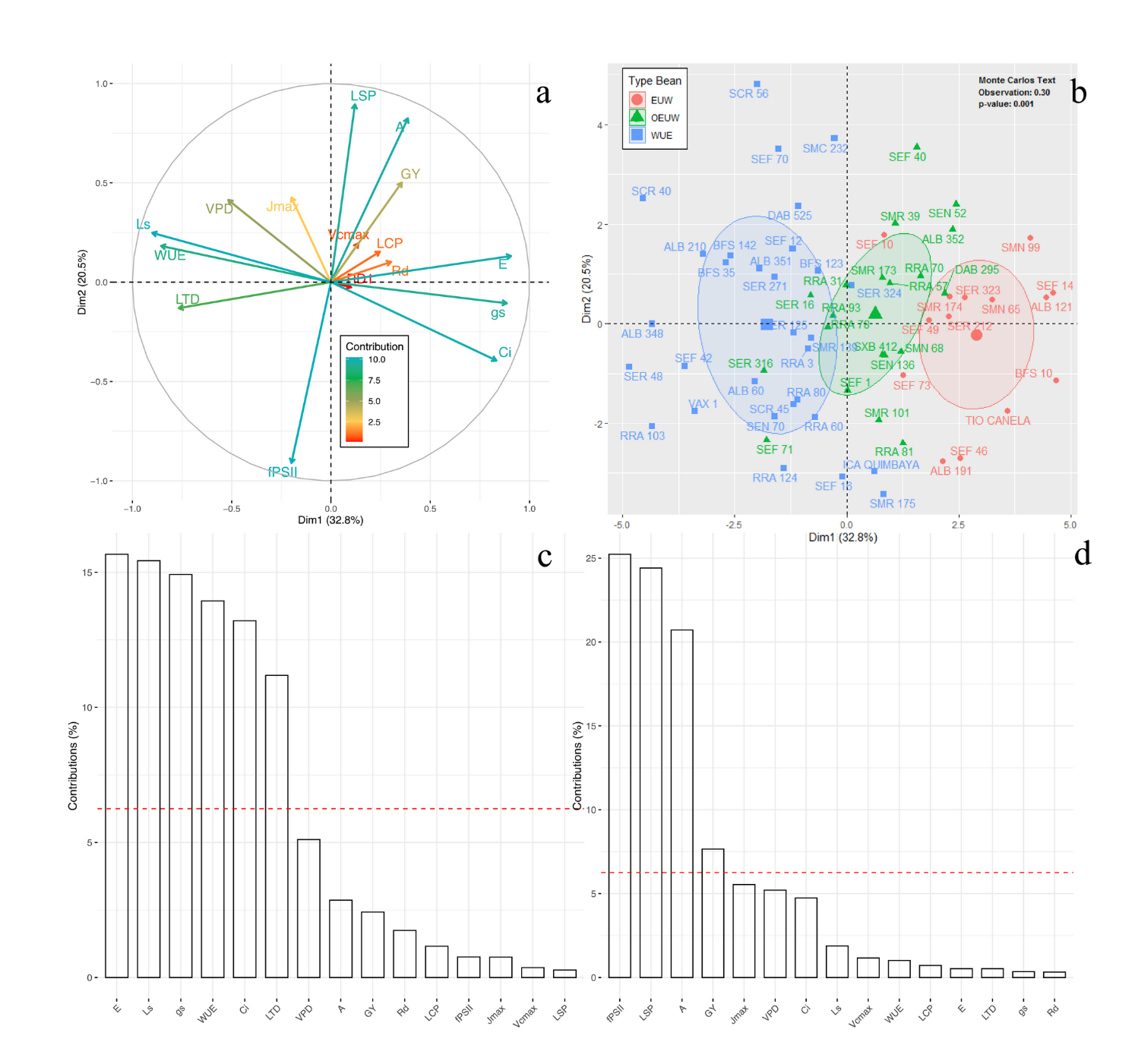
**

**Supplementary Figure 3.** Projection of physiological variables grouped by the different types of common bean genotypes grown at high temperatures, on the factorial plane F1/F2 of the PCA. (A) Correlation circle between physiological variables; (B) sorting of samples by common bean genotypes grown at high temperatures; (C) and (D) contribution of physiological variables to the formation of the F1/F2 principal components of the PCA under different types of common bean genotypes grown at high temperatures. *EUW*: Effective Use of Water, *WUE*: Water use efficient, OEUW: Opportunistically effective use of water. The acronyms are shown in Table 1 (main text).

**Supplementary Table 1.** List of common bean genotypes used in the study to assess heat tolerance

| **Genotype** | **Comercial class** | **Genepool** | **Growth habit** | **Classification** | **Cross** |
| --- | --- | --- | --- | --- | --- |
| ALB 121 | Brown | Mesoamerican | IIB | Inbred line | Pv x Pc - Mesos |
| ALB 191 | Black | Mesoamerican | IIB | Inbred line | Pv x Pc - Mesos |
| ALB 210 | Red | Mesoamerican | IIA | Inbred line | Pv x Pc - Mesos |
| ALB 348 | Red | Mesoamerican | IIA | Inbred line | Pv |
| ALB 351 | Red | Mesoamerican | IIA | Inbred line | Pv x Pc |
| ALB 352 | Red | Mesoamerican | IIB | Inbred line | Pv x Pc |
| ALB 60 | Red | Mesoamerican | IIB | Inbred line | Pv x Pc - Mesos |
| BFS 10 | Red | Mesoamerican | IIA | Inbred line | Pv |
| BFS 123 | Red | Mesoamerican | IIB | Inbred line | Pv x Pc |
| BFS 142 | Red | Mesoamerican | IIB | Inbred line | Pv x Pc |
| BFS 35 | Red | Mesoamerican | IIB | Inbred line | Pv |
| DAB 295 | Red mottled | Andean | I | Inbred line | Pv |
| DAB 525 | Red pink | Andean | I | Inbred line | Pv |
| ICA QUIMBAYA | Red | Andean | I | Inbred line | Pv |
| RRA 103 | Red | Andean | IIB | Inbred line | Pv x Pc |
| RRA 124 | Pink | Andean | IIB | Inbred line | Pv x Pc |
| RRA 3 | Cream striped pink | Andean | IIB | Inbred line | Pv x Pc |
| RRA 31 | Cream striped | Andean | IIB | Inbred line | Pv x Pc |
| RRA 57 | Red mottled | Andean | I | Inbred line | Pv x Pc |
| RRA 60 | Red mottled | Andean | IIA | Inbred line | Pv x Pc |
| RRA 69 | Pink | Andean | IIA | Inbred line | Pv x Pc |
| RRA 70 | Red | Andean | IIB | Inbred line | Pv x Pc |
| RRA 78 | Red | Andean | IIA | Inbred line | Pv x Pc |
| RRA 80 | Red | Andean | IIA | Inbred line | Pv x Pc |
| RRA 81 | Red | Andean | IIA | Inbred line | Pv x Pc |
| RRA 93 | Red | Andean | IIA | Inbred line | Pv x Pc |
| SCR 40 | Red | Mesoamerican | IIB | Inbred line | Pv |
| SCR 45 | Red | Mesoamerican | IIB | Inbred line | Pv |
| SCR 56 | Red | Mesoamerican | IIB | Inbred line | Pv |
| SEF 1 | Red | Mesoamerican | IIA | Inbred line | Pv x Pc x Pa |
| SEF 10 | Red | Mesoamerican | IIA | Inbred line | Pv x Pc x Pa |
| SEF 12 | Red | Mesoamerican | IIA | Inbred line | Pv x Pc x Pa |
| SEF 16 | Red | Mesoamerican | IIA | Inbred line | Pv x Pc x Pa |
| SEF 71 | Red | Mesoamerican | IIA | Inbred line | Pv x Pc x Pa |
| SEF 40 | Red | Mesoamerican | IIA | Inbred line | Pv x Pc x Pa |
| SEF 42 | Red | Mesoamerican | IIA | Inbred line | Pv x Pc x Pa |
| SEF 46 | Red | Mesoamerican | IIA | Inbred line | Pv x Pc x Pa |
| SEF 49 | Red | Mesoamerican | IIA | Inbred line | Pv x Pc x Pa |
| SEF 70 | Red | Mesoamerican | IIB | Inbred line | Pv x Pc x Pa |
| SEF 73 | Red | Mesoamerican | IIB | Inbred line | Pv x Pc x Pa |
| SEN 136 | Black | Mesoamerican | IIA | Inbred line | Pv |
| SEN 52 | Black | Mesoamerican | IIB | Inbred line | Pv |
| SEN 70 | Black | Mesoamerican | IIB | Inbred line | Pv |
| SER 125 | Red | Mesoamerican | IIB | Inbred line | Pv |
| SER 212 | Red | Mesoamerican | IIA | Inbred line | Pv x Pa |
| SER 271 | Red | Mesoamerican | IIA | Inbred line | Pv |
| SER 316 | Red | Mesoamerican | IIA | Inbred line | Pv |
| SER 323 | Red | Mesoamerican | IIA | Inbred line | Pv |
| SER 324 | Red | Mesoamerican | IIA | Inbred line | Pv |
| SER 48 | Red | Mesoamerican | IIB | Inbred line | Pv |
| SER 16 | Red | Mesoamerican | IIA | Inbred line | Pv |
| SMC 232 | Red mottled | Mesoamerican | IIB | Inbred line | Pv |
| SMN 65 | Black | Mesoamerican | IIB | Inbred line | Pv |
| SMN 68 | Black | Mesoamerican | IIB | Inbred line | Pv |
| SMN 99 | Black | Mesoamerican | IIB | Inbred line | Pv |
| SMR 101 | Red | Mesoamerican | IIB | Inbred line | Pv |
| SMR 139 | Red | Mesoamerican | IIB | Inbred line | Pv |
| SMR 173 | Red | Mesoamerican | IIB | Inbred line | Pv |
| SMR 174 | Red | Mesoamerican | IIB | Inbred line | Pv |
| SMR 175 | Red | Mesoamerican | IIB | Inbred line | Pv |
| SMR 39 | Red | Mesoamerican | IIA | Inbred line | Pv |
| SXB 412 | Cream | Mesoamerican | IIB | Inbred line | Pv |
| Tio Canela 75 | Red | Mesoamerican | IIA | Inbred line | Pv |
| VAX 1 | Cream striped | Mesoamerican | IIIB | Inbred line | Pv |

Abbreviations: Pv = *Phaseolus vulgaris*, Pa = *Phaseolus acutifolius,* Pc = *Phaseolus coccineus*.

**Supplementary Table 2.** The morphophysiological values of harvested plants (final harvest at physiological maturity) averaged from 3 replications (5 central plants from each replications). The three physiological groups (EUW, OWUE, WUE) are presented. The acronyms are the same as used throughout the study.

|  |  |  |  |  |  |  |  |  |  |  |
| --- | --- | --- | --- | --- | --- | --- | --- | --- | --- | --- |
| Trait | Units | EUW | | | OWUE | | | WUE | | |
| Pod | nb | 17.64 | ± | 1.84 | 16.60 | ± | 3.18 | 17.63 | ± | 2.12 |
| One Seed (DW) | g | 0.49 | ± | 0.03 | 0.40 | ± | 0.04 | 0.47 | ± | 0.02 |
| Seeds length | mm | 13.99 | ± | 0.44 | 13.83 | ± | 0.20 | 13.94 | ± | 0.39 |
| Seeds width | mm | 7.44 | ± | 0.30 | 7.07 | ± | 0.24 | 6.95 | ± | 0.19 |
| Seeds per pod | nb | 3.44 | ± | 0.29 | 2.79 | ± | 0.20 | 3.24 | ± | 0.24 |
| Non-viable seeds | nb | 1.91 | ± | 0.23 | 1.59 | ± | 0.26 | 1.77 | ± | 0.20 |
| Leaf (FW) | g | 64.90 | ± | 6.33 | 70.02 | ± | 5.83 | 73.57 | ± | 4.65 |
| Stem (FW) | g | 44.07 | ± | 2.66 | 40.30 | ± | 4.36 | 43.38 | ± | 3.79 |
| Root (FW) | g | 5.00 | ± | 0.32 | 4.44 | ± | 0.27 | 4.51 | ± | 0.33 |
| Pod (FW) | g | 25.72 | ± | 4.83 | 22.49 | ± | 6.81 | 24.97 | ± | 5.30 |
| Flowers (FW) | g | 0.68 | ± | 0.13 | 0.54 | ± | 0.11 | 0.45 | ± | 0.03 |
| Flower buds (FW) | g | 0.55 | ± | 0.14 | 0.47 | ± | 0.04 | 0.44 | ± | 0.04 |
